# Strategies to Maximize the Potential of Marine Biomaterials as a Platform for Cell Therapy

**DOI:** 10.3390/md14020029

**Published:** 2016-01-26

**Authors:** Hyeongmin Kim, Jaehwi Lee

**Affiliations:** 1 Pharmaceutical Formulation Design Laboratory, College of Pharmacy, Chung-Ang University, Seoul 156-756, Korea; hm.kim8905@gmail.com; 2 Bio-Integration Research Center for Nutra-Pharmaceutical Epigenetics, Chung-Ang University, Seoul 156-756, Korea

**Keywords:** marine biomaterials, cell therapy, tissue engineering, optimization, stimuli-responsive systems, delivery systems

## Abstract

Marine biopolymers have been explored as a promising cell therapy system for efficient cell delivery and tissue engineering. However, the marine biomaterial-based systems themselves have exhibited limited performance in terms of maintenance of cell viability and functions, promotion of cell proliferation and differentiation as well as cell delivery efficiency. Thus, numerous novel strategies have been devised to improve cell therapy outcomes. The strategies include optimization of physical and biochemical properties, provision of stimuli-responsive functions, and design of platforms for efficient cell delivery and tissue engineering. These approaches have demonstrated substantial improvement of therapeutic outcomes in a variety of research settings. In this review, therefore, research progress made with marine biomaterials as a platform for cell therapy is reported along with current research directions to further advance cell therapies as a tool to cure incurable diseases.

## 1. Introduction

When the tissues are damaged for a variety of reasons, the human body tries to recover the damaged portion of the tissues, but for most cases, these efforts result in tissue dysfunctionality and failure. In order to address these problems, numerous researchers have made many efforts to develop regenerative medicines, in particular, using therapeutic cells. The therapeutic cells including artificially cultured autologous or heterologous adult cells of specific tissues and stem cells derived from various sources have enormous potential to recover, repair and even replace the damaged or diseased tissues when they are properly processed, delivered or implanted to the targeted localities as a promising regenerative medicine. Indeed, the therapeutic cells have been used in regenerate diverse tissues such as heart, cartilage, bone, and cornea [1,2,3,4].

In the beginning, single cell or cell aggregate suspensions were directly injected to target localities without having delivery carriers. Although the injection methods showed modest therapeutic effects and have also been used in clinical practice, the therapeutic efficacies are frequently found to not be satisfactory and reproducible. The reason for this was largely because the injected cells were not retained in the target tissue, and most of them were washed out [5]. Furthermore, the injected cells typically cannot receive adequate physical and/or biochemical signals or supports from extracellular matrix (ECM), a complex where various proteins and polysaccharides such as collagen, hyaluronic acid, proteoglycans, and glycosaminoglycans are arranged. If the cells cannot receive appropriate supports and signals, they lose their viability, functions, and phenotype, and thereby this led to abnormal tissue formation or reduced therapeutic efficacies.

For this reason, the efficacy of cell therapy depends significantly on successful delivery of therapeutic cells to target tissues and localities, and surrounding microenvironment provided by the cellular carriers. To achieve the requisites for efficacious cell therapy, for the last decades, many efforts have been made to develop cell delivery systems using a variety of polymers. The cell delivery systems can incorporate therapeutic cells inside them, provide cell-friendly environments to the cells, be placed at the targeted site by proper external stimuli, and finally be degraded within the body, thereby delivering or integrating the cells to the targeted tissues. Besides such cell delivery systems, three-dimensional (3D) scaffolds have been used to grow therapeutic cells as an intact tissue to finally be engrafted to the body.

The platforms for cell delivery and tissue engineering must meet prerequisites as shown in Table 1. They should play pivotal roles as an artificial ECM providing a transient environment to support normal activities of cells such as adhesion to substrate, proliferation, and differentiation, and simultaneously exhibit appropriate properties including biodegradability and mechanical properties [6,7,8]. In particular, cell delivery systems have to deliver therapeutic cells to target sites with high efficiency, and scaffolds for tissue engineering should provide the initial structural support and allow the cells seeded within them to grow, metabolize, and product matrix, which are significantly important activities of the cells during the development of engineered tissues [9].

To date, diverse synthetic and natural polymers have been exploited to prepare such cell delivery systems and scaffolds. Synthetic polymers are well known for their processability and more precisely controllable physico-chemical properties, which are helpful for achieving reproducible mechanical and chemical properties and biodegradability, in particular with preferred uses of poly-α-hydroxy esters such as poly(lactic acid) (PLA) [33], poly(glycolic acid) (PGA) [34], and poly(lactic-co-glycolic acid) (PLGA) [35]. However, these polymers lack bioactivities for cell viability and growth and produce acidic byproducts on degradation, which is not desirable for fostering cell-friendly microenvironments [36]. In contrast, natural polymers inherently possess biological cues and often are similar to the natural ECM in terms of physical and chemical structure. For this reason, the biomaterials can promote cell adhesion to and spreading on substrate, as well as cell growth, and even differentiation. In addition, the biomaterials generally exhibit good biocompatibility and biodegradability, and can also be processed to preferred cell delivery vehicles such as hydrogels or hydrogel-based microspheres, films, and sponges under mild conditions. Thus, biomaterials have widely been investigated as a base material to prepare cell delivery systems and scaffolds for cell therapy applications.

**Figure 1:**
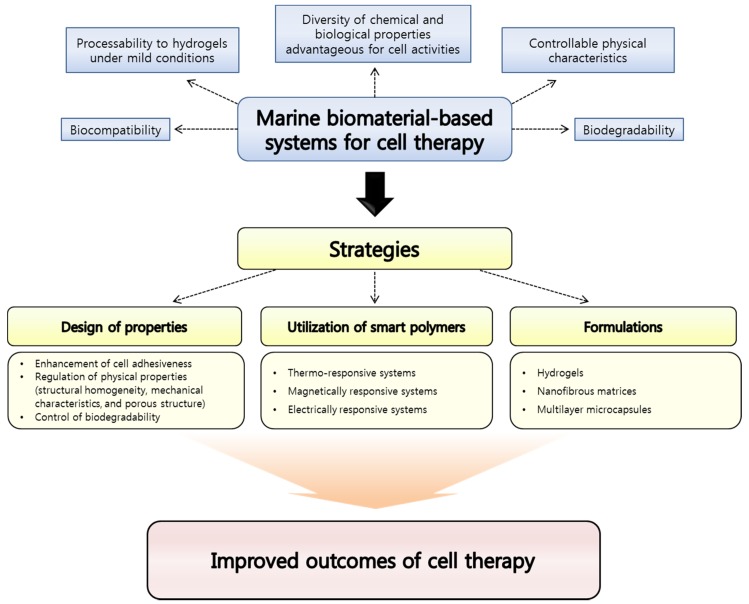
Advantages of marine biomaterials as a platform material for cell therapy applications and strategies suggested for improving outcomes.

Although they are still under-exploited resources, biomaterials derived from marine ecosystems are recently of enormous interest since the diversity of their chemical and biological properties is very attractive to cell therapy fields. In addition, the risk of causing toxicity is expected to be lower than synthetic polymers and even biopolymers found from other natural sources like mammalian alternatives [37]. To date, among various marine biomaterials, alginate and chitosan have extensively been investigated for cell therapy applications due to their biocompatibility, biodegradability, facile processability to hydrogels under mild aqueous conditions, and biological properties. Furthermore, other marine biomaterials such as carrageenans have also been increasingly investigated for cell therapy applications. However, for further efficient cell delivery and implantation, some properties of marine biomaterials have to be improved or additional functions need to be conferred to them. For this purpose, great efforts have been made to develop strategies for maximizing the potential of marine biomaterials as fundamental materials for fabricating platforms for cell delivery and tissue engineering as shown in Figure 1. In this review, representative marine biomaterials for cell therapy applications will be introduced, and the strategies devised until recently to enhance the utility of the marine biomaterials will be discussed.

**Table 1:** Suggested general requirements for marine biomaterial-based systems for successful cell therapy and suggested strategies.

Requirements	Description	Research Strategies	Reference
**Cell-friendly microenvironments**	Marine biomaterial-based systems should foster environments advantageous for cell activities such as proliferation and differentiation.	Enhancement of cell adhesiveness with cell-adhesive peptides	[10,11,12,13]
		Simulation of fibrous structure of natural extracellular matrix by preparing nanofibrous matrices	[14,15,16,17]
		Construction of smooth diffusive environment for gases and nutrients by controlling porous structures or volume of matrices	[18,19,20,21,22]
**Even cell distribution with minimizing stresses to cells**	In cell distribution process, cells should be uniformly distributed within matrices and not be largely damaged by stresses generated during the process such as shearing forces.	Regulation and optimization of rheological and mechanical properties with combined use of high and low molecular weight biomaterials	[23]
**Controllable biodegradability**	Marine biomaterial-based systems should be degraded with predictable rates in the body	Manipulation of biodegradability of biomaterials by chemical modification	[24,25,26]
**Minimally invasive implantability**	It is desirable that cell-incorporating systems are administered to the body with minimal invasiveness for patient convenience	Composite uses of biomaterials and thermo-responsive polymers	[27,28,29]
**Capability to deliver cells to target sites with high efficiency**	Therapeutic cells entrapped in cell delivery systems have to be selectively delivered to target sites with high efficiency.	Design of active cell delivery systems using magnetic particles	[30,31,32]

## 2. Representative Marine Biomaterials Exploited for Cell Therapy Applications

### 2.1. Alginate

**Figure 2:**
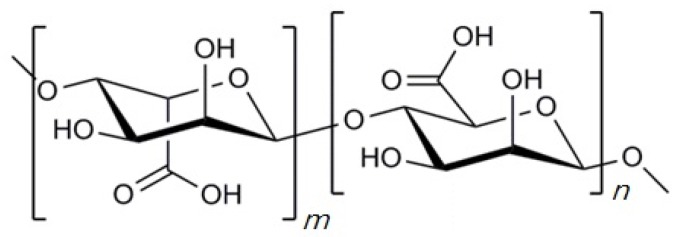
Chemical structure of alginate. “*m*” and “*n*” indicate the number of α-l-glucuronic acid and β-d-mannuronic acid residues, respectively.

Alginate is a natural occurring polysaccharide having blocks of (1-4)-linked β-d-mannuronic acid (M) and α-l-guluronic acid (G) monomers as shown in Figure 2, one of the most abundant biosynthesized materials in nature as a structural component in marine brown algae (*Phaeophyceae*) including *Laminaria hyperborean*, *Laminaria digitate*, *Laminaria japonica*, *Ascophyllum nodosum*, and *Macrocystis pyrifera* [38,39]. In general, the blocks are constituted by three different forms of polymer segment: consecutive M residues, consecutive G residues, and alternating MG residues [38]. The ratio of M residue and G residue varies depending on the natural source [6]. The length of each block can also be different according to the sources [40].

The polysaccharide has widely been utilized for a variety of applications as a biomaterial, in particular as the supporting matrix or delivery systems for tissue repair and regeneration, as it has desirable properties including biocompatibility, biodegradability, non-antigenicity, and hydrogel-forming ability from an aqueous solution under mild conditions. Alginate instantaneously forms hydrogels at pH > 6 by ionotropic gelation with divalent cations such as calcium, barium, or zinc ions. For the gelation procedure of alginate, only the G-blocks participate in intermolecular crosslinking with the divalent cations. Therefore, the ratio of M and G residues, sequence, length of G blocks, and molecular weight of the polymer affect the physical properties of the resultant hydrogels [41]. For this reason, it is important to consider the source of alginate and its chemical composition to use the polymer properly for each purpose as the physical properties of alginate significantly affect the phenotype and function of cells seeded in alginate hydrogels. On the other hand, alginate hydrogels can also be prepared by decreasing pH of alginate aqueous solutions to lower than the pK_a_ of the uronic acid residues at a controlled rate [42]. Through this procedure, hydrogen bonds can be formed between the alginate molecules largely due to the protonated carboxyl groups, followed by an intermolecular stabilization of the alginates. The resulting gel is frequently referred to as alginate acidic gels and they provide greater stability than ionically crosslinked alginate hydrogels. The greater stability of gel structures exhibited by the acidic gels has been attributed to the fact that the gel forming mechanism of the acidic gels does not primarily depend on crosslinking agents such as calcium ions that can leak into aqueous media. However, alginate acidic gels have generally demonstrated more brittle properties compared to the ionically crosslinked alginate gels. Thus, one can consider what types of gel forming mechanisms may be advantageous for specific purposes of research.

It is worth noting that alginate is a U.S. Food and Drug Administration (FDA)-approved polymer and this renders it a significantly important biomaterial for diverse applications such as tissue engineering and regeneration [43,44]. As it can easily be processed to various shapes/structures, alginate and its derivatives have extensively been used for fabricating the major biomaterial forms, e.g., hydrogels, microspheres, porous scaffolds, and nanofibers [38]. Owing to these advantages of alginate, it has extensively been studied as a representative marine biomaterial for cell therapy applications, and a variety of strategies for maximizing the potential of alginate have been devised. The strategies are discussed in detail below.

#### 2.1.1. Strategies for Designing Alginate-Based Systems for Cell Therapy Applications

##### Enhancement of Cell Adhesiveness Using RGD Peptides

Marine biomaterials have been modified to interact with cell receptors by conjugation of the corresponding ligands to them. In particular, this strategy has extensively been applied to alginate as the negatively charged marine biomaterial in an aqueous solution state inherently lacks the cell adhesiveness. The most commonly utilized ligand for cell anchoring is arginine-glycine-aspartic acid (Arg-Gly-Asp, RGD) sequences. These RGD amino acid sequences can be chemically coupled with alginate using a water-soluble carbodiimide chemistry [45,46,47]. The RGD sequence is one of the most physiologically ubiquitous binding ligands derived from the ECM proteins including fibronectin, collagen, and laminin [48,49]. Cellular integrins recognize the RGD sequence and link the intracellular cytoskeleton with the ECM. By this process, cells receive signals needed for cell survival and proliferation. In addition, the RGD sequence prevents cell apoptosis. The signals propagated from the cell–matrix adhesion are known to activate various signaling pathways contributing to the suppression of apoptosis. Without these signals, anoikis, a form of programmed cell death induced by detachment of anchorage-dependent cells from the surrounding matrix, can occur [50,51]. Thus, the presence of such sequences for cell adhesion to the surrounding matrices is essential for cellular viability and activities.

There are numerous research articles on applications of the cell crosslinking strategy to cell delivery systems or scaffolds composed of alginate for cell therapy applications. For example, Shachar *et al.* investigated the effect of immobilized RGD peptide in alginate scaffolds for cardiac tissue engineering [10]. They immobilized the RGD peptide to sodium alginate using an aqueous carbodiimide chemistry, followed by seeding cardiomyocytes within the scaffolds. The presence of the RGD peptide sequence was found to promote *in vitro* cardiac tissue regeneration and demonstrated a better preservation of the tissue formed. The cardiomyocytes seeded within the scaffolds were able to reorganize their myofibrils and reconstruct myofibers with a typical myofiber bundle with expression of the relevant proteins such as α-actinin, *N*-cadherin, and connxin-43. In addition, the non-myocyte cells seeded within the scaffolds along with the cardiomyocytes exhibited stretched, star-like morphologies, implying successful anchoring and spreading. In contrast, the cells cultured in the unmodified alginate scaffolds were round in shape, lacked the typical striation of cardiac muscle tissue and showed decreased levels of the protein expression, corroborating the importance of the existence of the RGD sequences.

More recently, alginate grafted with the RGD peptide sequence and at the same time compositionally modified was reported by Sandvig *et al.* [11]. In the study, the proportion of M- and G-sequences within the alginate chemical structure was controlled to tailor its physical properties along with conferring the biomaterial cell adhesive property using the RGD peptide. They coupled mannuronan, poly-β-(1→4)-d-mannuronate, with the RGD peptide sequence using a carbodiimide chemistry, and epimerized the peptide-coupled mannuronans with the mannuronan C-5 epimerases, thereby introducing G- and MG-blocks into their chemical structure. By this way, the peptide sequence coupled to the M-units does not interfere with G-blocks that primarily contribute to the hydrogel formation. Then, they immobilized olfactory ensheathing cells (OECs), a promising candidate cell type in transplant-mediated CNS repair, to the hydrogels and the microbeads composed of the modified alginate described above. As a consequence, the authors could produce alginate hydrogels with different contents of G-blocks and resulting varying physical properties, and confirmed that OECs seeded within the alginate gels formed large clusters of rounded cells with bipolar protrusions. The cells also exhibited higher viability than those cultured in unmodified alginate hydrogels. These studies together suggest the introduction of the peptide sequences for cell adhesion is a promising strategy for maximizing the potential of alginate as a biomaterial for tissue engineering applications.

##### Control of Structural Homogeneity by Modifying Crosslinking Densities

Ionic marine biopolymers such as alginate (anionic) and chitosan (cationic) can be physically crosslinked using ionic crosslinking agents. The most noteworthy advantage of the ionic crosslinking method for preparing alginate hydrogels is this crosslinking method does not require any organic solvents, and the crosslinking process is performed under gentle conditions for the entrapped therapeutic cells [52]. As for alginate, the most common method to fabricate hydrogels is to crosslink the alginate with divalent cations. The divalent cations interact with blocks of G monomers of alginate to form ionic bridges, forming an “egg-box” structure and leading to the resulting gelation of alginate [39].

Among the cations used as an ionic crosslinking agent for the gelation of alginate such as calcium, magnesium, and barium ions, calcium ions have most widely been used. [52,53]. In particular, calcium chloride has most frequently been utilized as an ionic crosslinking agent in external gelation methods for preparing alginate hydrogels because the alginate crosslinking process using the calcium salt is very simple and provides immediate and non-toxic cell entrapment [6]. In practice, this gelation method has extensively been harnessed for tissue engineering applications, e.g., bone, cartilage, intervertebral disk, and adipose tissue [54,55,56,57]. Nonetheless, due to its too fast crosslinking reaction rate, unbalanced crosslinking density through alginate hydrogels formed and a polymer concentration gradient within the gel can occur [52]. This non-homogeneous crosslinking density may limit the usefulness of the alginate hydrogels for cell therapy applications as it does not provide structural uniformity of the hydrogels that is significantly important for even cell distribution and well-controlled mechanical properties. Furthermore, the fast gelation process by calcium chloride limits the application of alginate on injectable cell delivery systems or scaffolds.

In this context, Kuo *et al.* devised an internal gelation method that controls the gelation process more precisely using calcium salts with low aqueous solubility such as calcium carbonate [52]. Calcium carbonate exhibits low solubility in pure water at neutral pH, but soluble at acidic conditions, thereby allowing its homogenous distribution in the alginate solution prior to gelation [52,58]. Free calcium ions are then released from the calcium salts by decreasing the pH, generally using glucono-δ-lactone (GDL), thereby triggering gradual gelation. Indeed, they demonstrated that alginate hydrogels with homogeneous crosslinking density and uniform mechanical properties can be fabricated by the gelation method using calcium carbonate. Furthermore, the mechanical properties of alginate hydrogels prepared using calcium carbonate were stronger than those fabricated only using calcium sulfate, and were more soluble in aqueous media than calcium carbonate. The gelation process also can become faster by a combined use of calcium carbonate and calcium sulfate maintaining uniform crosslinking density and mechanical characteristics. From the histological experiments of the study, it was demonstrated that the cells were uniformly distributed in the alginate hydrogels prepared using calcium carbonate as a crosslinking agent. Thus, the ionic crosslinking method using a calcium salt with low aqueous solubility is a promising strategy for preparing alginate hydrogels with homogeneous properties. Although the internal gelation method has not been extensively explored compared to the external gelation method, recently, it has been increasingly studied to prepare alginate cell delivery systems for tissue engineering [59,60,61].

##### Regulation and Optimization of Physical Properties and Biodegradability

Hydrogels prepared with alginate of high molecular weight (HMW) show stronger and useful mechanical properties than those fabricated with biomaterial of low molecular weight (LMW). However, solutions of the HMW polymer can be too viscous, and this phenomenon is often problematic in terms of cell distribution in the solutions [62]. The therapeutic cells suspended in the viscous polymer solutions are also likely damaged by high shear forces generated during mixing and injection processes, leading to decreased therapeutic efficacy of the cells [63]. Thus, there have been needs to decouple the relationship between mechanical properties and viscosity of the alginate solutions for establishing optimized physical properties of alginate hydrogels and incorporating therapeutic cells without undesirable effects on them simultaneously.

To achieve a breakthrough to address this problem, a strategy of manipulating the molecular weight of alginate and its distribution has been devised by Kong *et al.* [23]. They used HMW alginate rich in GG-blocks and (LMW) alginate made by γ-irradiating the HMW polymer. The length of GG-blocks of the LMW alginate was maintained as that of the HMW alginate as the γ-irradiation could break down MG-blocks and MM-blocks selectively due to the lower stiffness of the blocks than that of GG-blocks [64]. The HMW alginate contributes mainly to increasing the viscosity of the polymer solution as they are entangled each other, thereby increasing the relaxation. In contrast, the LMW alginate can avoid the strong physical interactions occurred between the HMW alginate molecules and accordingly did not increase the viscosity of the solution significantly. However, the hydrogels prepared with the LMW alginate could possess appropriate mechanical properties because the LMW alginate retained a capability to form mechanically strong hydrogels due to the conserved GG-blocks after the γ-irradiation process. With these principles, alginate solutions exhibit low viscosity before gelation, facilitating uniform cell distribution without significant physical damage to the cells, and hydrogels prepared with them possess proper mechanical characteristics. Thus, this strategy can be useful to independently control the viscosity of alginate solutions and the mechanical properties of its hydrogel.

As for biodegradability of alginate, it is inherently non-biodegradable in mammals as they lack enzymes cleaving the polymer chains. Although alginate hydrogels crosslinked by calcium cations can be dissolved by the exchange of the divalent ions with monovalent ions existing in the surrounding media, many commercially available alginates cannot be completely removed from the body. The reason for this is because molecular sizes of the alginates are greater than the molecular weight limits of the renal clearance [65]. Thus, dissolution rates of alginate hydrogels have typically been controlled by varying concentrations and molecular weights of the biomaterial used. However, both of the factors significantly affect the mechanical properties of the alginate hydrogels, thereby limiting the controllability of dissolution rates of the hydrogels.

As a strategy to render alginate biodegradable, alginates have been partially oxidized. Sodium periodate has typically been utilized to oxidize alginate. The oxidation by sodium periodate cleaves the carbon–carbon bond of *cis*-diol group in the urinate residue, thereby enabling the oxidized alginate to be degraded [6]. The oxidation of alginate does not impair the capability of the polymer to form gels in the presence of divalent cations [6]. As such, hydrogels prepared with the oxidized alginates have demonstrated the promise as cell delivery systems and scaffolds for various cell therapy applications.

Beyond only rendering alginate biodegradable, many efforts have been made to decouple the interdependence between the biodegradability and mechanical properties of alginate hydrogels [24,25,26]. For example, Lee *et al.* prepared hydrogels with poly(aldehyde guluronate) (PAG) composed of only β-d-guluronate residues covalently crosslinked using adipic acid dihydrazide (AAD) [24]. By the crosslinking reaction, hydrazone bonds labile to hydrolysis are formed. In the study, despite the LMW of PAG (5,700 Da) and the low crosslinking density, the hydrogels exhibited a retarded degradation behavior despite their low crosslinking density. This property of PAG hydrogels goes against the common relationship between degradation rate and crosslinking density of polymer hydrogels. The mechanism for this was that the large number of single-end ADD molecules, caused by the high concentration of ADD, allowed re-cross-linking of PAG strand when the hydrazine bonds in the crosslinked polymers were hydrolyzed, thereby delaying their degradation. These results demonstrated the possibility that alginate hydrogels with weak mechanical properties can possess slow biodegradation rates.

There is another study on alginate hydrogels of which mechanical properties and degradation rates are independently controlled [25]. In the study, alginate hydrogels composed of two partially oxidized alginates having a two-fold difference in molecular weight were investigated. The LMW alginate was produced from the HMW alginate using a method that does not change the length of G-blocks, which exclusively contribute to the gelation of alginate. When preparing alginate hydrogels using only the HMW alginate, although the hydrolytic chain breakage occurred, it did not cause complete separation of the chains due to the intrinsic chemical structure of the HMW alginate. In contrast, for the hydrogels fabricated with both the HMW and LMW alginates, the complete chain breakage was achieved quickly because the presence of the LMW alginate in the hydrogels made the separation of the polymer chains easier. In addition, the G-blocks of the LMW alginate having the same length to that of the high MW alginate contributed to retaining the mechanical properties of the hydrogels. Likewise, *in vivo* study, the binary oxidized gels injected into mice were degraded more rapidly than the gels prepared only with the high MW alginate, leading to more successful formation of bone tissue, as characterized by more cells, matrix, and tissue resembling bone in tissue sections.

Another report has suggested a different strategy of using alginates having different length of G blocks in the polymer chains [26]. Owing to the difference in the length of G-blocks, the size mismatch between ionically crosslinking blocks in polymer chains occurred, and this phenomenon promoted the exchange process between the divalent cations and the monovalent cations present in the surrounding medium. This is probably due to a crosslinking junction formed in the gel above which prevented the carboxylic acids from participating in the crosslinking, thereby increasing the hydrophilic property of the junction. In this situation, the monovalent cations can replace the divalent cations maintaining the crosslinking state of the polymer chains more easily, resulting in the dissociation between the G blocks. However, there was no significant decrease in the elastic modulus and swelling ratio, indicating the size mismatch between G-blocks in polymer chains did not affect the mechanical properties largely. They also showed the improved cartilage tissue formation *in vivo* with the alginate having different length of G-blocks. These studies together suggest alginate hydrogels capable of independently controlling their biodegradation rate and mechanical properties are promising for obtaining better cell therapy results.

#### 2.1.2. Stimuli-Responsive Alginate Systems for Cell Therapy Applications

Stimuli-responsive polymeric systems indicate polymer-based formulations that can react to small changes in the environment such as pH, temperature, and magnetic or electrical signals, thereby varying their properties in a multidirectional way. Such sensitivity to external stimuli makes the polymeric material-based systems cope with various situations in a way to achieve their ultimate goal effectively. Due to their versatility, stimuli-responsive polymers have increasingly been exploited in the biomedical field. In particular, many researchers in the cell therapy field have used biocompatible materials and stimuli-responsive polymers compositely as such approaches provide outstanding performance without having undesirable effects on cells or host. In this context, as biocompatible and biologically active materials, marine biomaterials such as alginate and chitosan have actively been explored with stimuli-responsive polymers for cell therapy applications. In this section, some important cases of alginate-based stimuli-responsive systems that have shown successful outcomes in cell therapy field are discussed.

##### Thermo-Responsive Alginate Systems

Thermo-responsive polymers can be chemically grafted onto marine biopolymers or physically mixed with the polymers such as alginate and chitosan, thereby conferring thermo-responsiveness on the marine biopolymers or the hydrogels composed of the polymers [27]. There are two thermo-responsive polymers primarily preferred for cell therapy purposes: poly(*N*-isopropylacrylamide) (PNIPAAm) and Pluronic F127. PNIPAAm is a well-known thermo-responsive polymer. Gelation of this polymer solution occurs when the temperature increase above the lower critical solution temperature (LCST) [66]. The mechanism of the phase transition of PNIPAAm by temperature changes is based on thermally induced release of water molecules bound to the isopropyl side groups of the polymer structure above its LCST, which leads to enhancement of hydrophobic interactions between isopropyl groups [28,67]. Pluronic F127 is also used to design thermo-sensitive marine biopolymer hydrogels. Pluronic F127 is a thermo-responsive triblock copolymer consisting of a central hydrophobic block of polypropylene oxide (PPO) franked by two hydrophilic blocks of polyethylene oxide (PEO). It is thus hydrophilic and can be used as a non-ionic surfactant. The thermo-responsive property of this polymer depends on its molecular weight and ratio between PEO and PPO [68]. Similar to PNIPAAm, temperature-dependent change in the structure of water surrounding the PPO blocks contributes the gelation behavior of Pluronic F127 [69,70]. Pluronic F127 has been shown to be non-irritant and cytocompatible with a variety of cell types [71,72]. The polymer is regarded as safe and one of the very few synthetic polymers approved by the FDA for clinical applications [73,74].

The thermo-responsive hydrogels can be harnessed as injectable hydrogels and cell culturing platforms. For injectable hydrogels, they can be administered to the body with minimal invasiveness and possess advantages such as site specific introduction, cost effectiveness, and patient convenience [75,76]. On the other hand, by using the cell culturing systems composed of marine biopolymers modified with thermo-responsive polymers, the cultured cells can be harvested as an intact state, *i.e.*, maintaining the ECM as trypsin treatment is not necessary during the harvesting process [67]. By combining the advantages of marine biopolymers such as biocompatibility, biodegradability, and various biochemical activities, and the thermo-sensitive polymers, one can fabricate the scaffolds or cell vehicle systems with promising functionalities for cell culture and delivery.

For instance, Abdi *et al.* modified alginate with Pluronic F127 for cell injection applications [27]. They designed a blended hydrogel composed of alginate, Pluronic F127, and hyaluronic acid to combine benefits of the natural biomaterials and the thermo-responsive polymer. By blending the polymers, *in situ* characteristics and the native microenvironment for maintaining cell functions were simultaneously achieved. The hydrogels were prepared using aqueous solvents under mild temperature conditions, minimizing undesirable effects on the functions and viability of skeletal muscle cells entrapped within. The composite hydrogels showed flow behavior under non-physiological temperature (22 °C) and formed gels at the physiological temperature (37 °C). When the skeletal muscle cells were cultured on the Pluronic F127 hydrogels, 60% of viability was achieved. However, in the hydrogels composed of Pluronic F127 and alginate, the cell viability was increased to 80% probably due to the biochemical cues of alginate exposed to the cells cultured. In addition, in the case of the hydrogels prepared with Pluronic F127, alginate, and hyaluronic acid, they demonstrated excellent biocompatibility with almost 100% cell viability. The reason for this might be because hyaluronic acid, the main component of ECM, provided a more favorable environment to the cells, thereby supporting cell growth and proliferation [77,78]. When the polymer–cell mixture was injected to BALB/c nu/nu mice, the gel formation was clearly observed on the skin of each nude-mouse. Furthermore, muscle tissue-like mass and some sign of vascularization were also observed, indicating the bio-functionality of the hydrogels could promote the cellular activities. H & E staining also demonstrated the adhesion and spreading of the cells inside the hydrogels.

Tan *et al.* also developed thermo-sensitive alginate-based injectable hydrogels [29]. They synthesized a thermo-sensitive comb-like polymer with alginate as the backbone and PNIPAAm as pendant group by coupling carboxylic end-capped PNIPAAm to aminated alginate through amide bond linkages with purposes to improve controllability of degradation rates and develop cytocompatible and injectable hydrogels for cell therapy applications. As a result, hydrogels composed of aminated alginate-g-PNIPAAm (AAlg-g-PNIPAAm) exhibited sol-gel phase transition behavior when they were evaluated rheologically. The viscosity of the hydrogels was decreased as temperature was increased from 25 to 37 °C. In particular, from 34 to 36 °C, the viscosity of the hydrogels increased dramatically since the aqueous solution of the AAlg-g-PNIPAAm transformed to elastic hydrogels. The AAlg-g-PNIPAAm hydrogels also showed a controllable degradation rate. The degradation rate of the hydrogels increased with increasing the portion of PNIPAAm grafted at 37 °C, implying the PNIPAAm modification can be utilized to improve the controllability of alginate hydrogels. In terms of cell distribution and cytocompatibility within AAlg-g-PNIPAAm hydrogels, human bone mesenchymal stem cells (hBMSCs) could be uniformly distributed, survive well and proliferate in the hydrogels during the culture period. Moreover, the thermo-sensitive alginate hydrogels preserved the cell viability better than the unmodified alginate hydrogel. The reason for this might be because the microstructure and high water content of AAlg-g-PNIPAAm hydrogels were very similar to the natural ECM of tissues, thereby supporting cell survival and proliferation. Together, the studies indicate that thermo-responsive alginate hydrogels have great potential in cell therapy applications.

##### Magnetically Responsive Alginate Systems

Although cellular delivery systems composed of marine biomaterials have extensively been studied, in most cases, cell release and delivery from those systems have depended only on passive cell diffusion, natural cell migration, and biomaterial degradation. In such manner, on-demand cell release or cellular delivery to selective localities can hardly be achieved. For this reason, novel strategies for the active cell delivery have increasingly been developed. Among the recently devised strategies for efficient cell transport, magnetic cellular delivery systems have shown great promise.

For the last several years, hydrogels consisting of alginate and iron oxide particles, called also ferrogels, have been investigated for on-demand cell delivery purposes [30,31]. Ferrogels are magnetic field responsive hydrogels and typically consist of a polymer matrix incorporating iron oxide particles, commonly magnetite or maghemite. The hydrogels can be deformed remotely under the application of a non-uniform external magnetic field, thereby releasing drug, bioactive materials, and cells in a non-invasive and precisely timed manner.

For instance, Zhao *et al.* developed deformable ferrogels under magnetic field, thereby releasing therapeutic cells entrapped within the gels [30]. They fabricated macroporous RGD-modified alginate hydrogels incorporating the magnetic particles. Under the influence of non-uniform external magnetic field, a body force is applied to the ferrogels proportionally to the gradient of the magnetic force, leading to the deformation of the gels [79,80]. The therapeutic cells entrapped within the ferrogels are then released from the gels probably by the water convection and associated shear forces generated during the deformation procedure of the gels’ 3D structure. Indeed, they seeded human dermal fibroblasts within the ferrogels prepared in the study, and it was found that the magnetic stimulation promoted the cell release from the gels successfully both *in vitro* and *in vivo* model. In addition, the cell release could be controlled by varying the RGD density on the surface of the ferrogels. The lower RGD densities provide weaker cell adhesion to the ferrogels, leading to more facile cell detachment and resulting in fast release from the gels [81]. The cell release can also be controlled by other parameters such as the strength of applied magnetic field, number of magnetic cycles, and frequency of magnetic stimulation. In terms of the viability and functionality of the cells entrapped within and released from the magnetic hydrogels, it was found that more than 95% of the cells released were viable and proliferated successfully to a confluent state. Thus, they demonstrated the promise of alginate-based hydrogels incorporating magnetic particles and modified with the cell adhesion amino acid sequences for on-demand and active cell delivery.

However, when the alginate-based ferrogels are scaled down to smaller sizes to meet some specific conditions, it is challenging to achieve the deformation of the gels enough to release the therapeutic cells due to: (i) the reduced amount of iron oxide particles available; (ii) the reduced magnetic field gradient applied to the magnetic particles; and (iii) the reduced pore size associated with the scale down of the ferrogels. Thus, to deform the ferrogels with smaller sizes significantly, the content of iron oxide within the gels needs to be increased. In this case, however, the risk of toxicity generated by the iron oxide particles is also increased, which are undesirable for the cell delivery purposes [82]. Moreover, the increased amount of the iron oxide particles can render the ferrogels stiffer than intended, potentially resulting in unwanted changes in cell fate [83,84]. To address these problems, Cezar *et al.* developed biphasic ferrogels where the iron oxide particles are distributed at one side of the gels [31]. To prepare the biphasic ferrogels, the alginate solution containing iron oxide particles was subjected to a vertical magnetic field gradient during polymerization procedure. As the resulting biphasic ferrogels possess macroporous structure and simultaneously sufficient amounts of iron oxide particles to induce the deformation of the gels, they could release cells entrapped within them and maintain softness. As a matter of fact, the biphasic ferrogels demonstrated a 2.4-fold increased deformation compared to monophasic ferrogels containing the same amount of iron oxide particles. Thus, the problems that occurred with the miniaturization of the ferrogels could be addressed.

On the other hand, Janus alginate hydrogel particles were developed by Zhao *et al.* to reduce undesirable interactions between therapeutic cells and magnetic particles encapsulated in the state of being encapsulated [32]. Janus particles are anisotropic particles having distinct compositions in each hemisphere [85,86]. One can confer Janus particles diverse functions by altering the asymmetric property. In the study, cells and magnetic nanoparticles could be encapsulated in each hemisphere of the Janus alginate hydrogels’ particles using a microfluidic technique. They demonstrated the possibility of remote control over the Janus alginate particles under the application of external magnetic field by using a microchip with the nickel array. In addition, the Janus alginate particles showed non-significant toxicity to the encapsulated cells with about 10% lower viability than those in ordinary alginate hydrogel sheets. The Janus alginate particles thus can be potentially used as a carrier of therapeutic cells for a variety of applications such as targeted delivery based on magnetic control. Thus, the magnetically responsive alginate is a promising candidate for active and targeted cell delivery.

#### 2.1.3. Platforms for Cell Delivery and Tissue Engineering

##### Hydrogels

Hydrogels are generally composed of hydrophilic polymers with three-dimensionally cross-linked networks and high water contents [66,78]. Hydrogels have widely been exploited in the tissue engineering and regenerative medicine fields because they have various advantageous properties for incorporating the therapeutic cells. Hydrogels can typically be prepared in mild processing conditions and offer cell-friendly environments to the encapsulated cells. In addition, they present high water content, soft and viscoelastic properties, and functional and structural similarities to the ECM of human tissues [66,87]. In particular, hydrogels consisting of natural biopolymers can be recognized by the biological environment as biological macromolecules [88]. In the last decades, alginate has extensively been used to prepare hydrogels for cell therapy applications as the biomaterial is biocompatible and can be fabricated to hydrogels under a mild gelation process. Nonetheless, conventional alginate hydrogels do not meet all the conditions required for successful cell delivery and tissue regenerations. In this context, a variety of approaches for maximizing the potential of alginate hydrogels for cell therapy have been devised.

For instance, alginate hydrogels have been modified to be injectable. In general, injectable hydrogel compositions are in the state of solutions prior to administration and implantation to the body, while conventional hydrogel formulations are pre-gelled before use. Injectable hydrogels provide several advantages over preformed hydrogel-based approaches for clinical applications. The injectable hydrogel systems are liquid state before injection to the human body, but they become hydrogels under the physiological environment. They can thus be transferred to the body with minimally invasive methodologies, thereby augmenting patient comfort and leading to faster recovery and lower healthcare costs [89]. Other than these features, the injectable hydrogel systems provide several additional advantages: (i) facile distribution of therapeutic cells within the hydrogels; (ii) simple procedure of injection to the body; (iii) adaptable filling of defects present around in the tissues of interest; (iv) site specific delivery of the cells [90,91,92,93]. Owing to these benefits, the injectable hydrogels have widely been studied as cell carriers for *in vivo* tissue engineering, and also regarded as one of the most ideal cell delivery systems for clinical applications.

To date, naturally derived polymers have extensively been studied as fundamental materials for preparing injectable hydrogels for cell therapy applications because their chemical structures are analogous to the natural ECM, thereby offering cell-friendly environments with a variety of biochemical signals. Understandably, the marine biomaterials such as alginate have been exploited to fabricate injectable hydrogels since they also provide biological signals necessary for maintaining normal cellular activities. Due to these advantages of the marine biopolymers, they have extensively been studied as base materials to prepare injectable hydrogels for cell therapy applications. For alginate, for example, Kim *et al.* demonstrated injectable hydrogels as a suitable delivery system for human adipose-derived stem cells (hADSCs) to engineer adipose tissue [94]. The study was performed to overcome an obstacle that the adipocytes differentiated from hADSCs can readily spread from the recipient site, which prevents organizing and constructing the adipose tissues from the cells, unless using an appropriate cellular carrier. To fabricate the injectable alginate hydrogels for the adipose tissue engineering application, an internal gelation strategy was utilized [52,59]. They used HMW alginate and LMW alginate obtained by γ-irradiating the HMW alginate. The two types of alginate were then oxidized by treatment with sodium periodate to confer the polysaccharides’ biodegradability by enzymes, followed by conjugating peptide sequences for cell adhesion using a carbodiimide chemistry. The modified alginate solutions containing pre-differentiated cells from the hADSCs were successfully gelled by aqueous slurry of calcium sulfate after being injected into the abdomen of each male nude mice. As a consequence, the gel formed did not migrate away from the injection location, or invade the nearby tissues. There were no any undesirable symptoms such as inflammation, swelling, or redness. At the time of harvesting the newly formed tissue, a well-organized adipose tissue was observed with evidence of neovascularization, while the cells injected without gel carrier (control) showed no tissue formation at sacrifice. In addition, expression of markers for the adipose tissue was detected such as peroxisome proliferator-activated receptor-gamma and adiponectin. Thus, the injectable alginate hydrogels demonstrated potential for engineering the adipose tissues with their intrinsic characteristics.

Bidarra *et al.* also studied injectable alginate hydrogels for the delivery of endothelial cells [93]. They also prepared the injectable alginate matrix using HMW and LMW alginate oxidized with sodium periodate and grafted with peptide sequences for cell adhesion. Calcium carbonate was used for gelation of the modified alginate solution, and the gelling process triggered by glucono-δ-lactone added to decrease pH, thereby causing the calcium carbonate to dissolve under the simulated physiological conditions examined. Human umbilical vein endothelial cells (HUVECs) then were cultured within the alginate hydrogels. As a result, the alginate hydrogels demonstrated the contour-adaptability when casted in different molds. The alginate matrix modified with the RGD sequences also showed better cytocompatibility compared to unmodified alginate hydrogels. In addition, expression of angiopoietin-2, a family of angiogenic modulators almost exclusively produced by endothelial cells, was detected by an RT-qPCR assay, substantiating the functionality of the cells cultured. Combining these results, the injectable alginate hydrogel was found to be promising as a vehicle for vascular cell-based therapy application.

Different from the aforementioned injectable alginate hydrogels prepared with internal gelation methods using calcium cations for alginate crosslinking, covalently crosslinked shape-memory alginate hydrogels also have been developed as injectable hydrogel systems for cell therapy [95,96]. Thornton *et al.* demonstrated the potential of the shape-memorizing alginate hydrogels as a bulking agent that can be injected with minimal invasiveness [95]. The hydrogels were prepared by standard carbodiimide chemistry using 1-ethyl-(dimethyl aminopropyl) carbodiimide, 1-hydroxybenzotriazole and the bifunctional cross-linker AAD [97]. They could be formed into various 3D shapes such as disc, rectangle, and triangle and the shapes retained their geometry after the lyophilization process. The hydrogels freeze-dried were then physically compressed to a cylindrical form and regained their initial shape after being exposed to water of PBS, indicating their shape memorizing ability. These shape memorizing characteristics of the hydrogels were also observed in *in vivo* study. When retrieving the shape memorizing scaffolds, they displayed high fidelity with regard to shape recovery and maintenance *in vivo*. In contrast, alginate implants crosslinked with calcium ions showed irregular shape. In terms of cell infiltration into the scaffolds and proliferation, mature fibrous tissue was observed in the injectable alginate matrix, exhibiting fibroblasts, ECM, and neo-vascular tissues. However, cell migration was only observed between the fragments of calcium alginate gel and the cells did not infiltrate the gel. Combining these results together, the covalently crosslinked alginate hydrogels having shape retaining ability have the potential as an injectable hydrogel scaffolds as they provide adequate 3D environments to the cells for infiltration into the gels and organization of tissues by retaining their shape and porous structure as well as their delivery procedure in a minimally invasive manner.

More recently, Wang *et al.* reported advanced shape-memory alginate systems for cell and growth factor delivery [96]. In addition to the shape recovering ability, their injectable alginate scaffolds was designed to have biodegradability that can be adjusted according to the same time frame as new tissue formation, a surface structure to support optimal affinity of seeded cells, and capacity to release growth factors to promote tissue regeneration. To achieve these characteristics, they used LMW and HMW alginate oxidized for increasing susceptibility to hydrolysis and modified with RGD sequences to fabricate the injectable alginate scaffolds, and then incorporated insulin-like growth factor-1 (IGF-1) into the scaffolds. The alginate scaffolds after rehydration exhibited good shape memorizing ability evaluated with swelling ratio and porosity. Moreover, mouse myoblasts used as model cells were found to reside not only on the surface of the scaffold but also inside the scaffold at least 100 μm depth and also formed cell clusters, implying the scaffolds provided a proper 3D structure for cell infiltration into the matrix and proliferation. For biodegradation rate, it could be controlled depending on the oxidation degree of the alginate. The incorporated IGF-1 also was observed to be released from the scaffolds in a sustained manner for approximately two weeks. Overall, this study demonstrated the potential of the multifunctional injectable scaffold for tissue engineering applications.

##### Nanofibrous Matrices

Collagen is one of the major components of ECM and has a fibrous structure with fibrils of varying diameters (50–500 nm). This component of ECM affects cell behaviors by facilitating cell attachment to the matrix of fibrous structures and promoting cellular activities. The cells attached to the matrix can retain their normal phenotype and grow along the fiber orientation. Moreover, the collagen fibrils contribute to retain the mechanical resistance of some tissues including the skin. Thus, the existence of the ECM’s fibrous structure is critical for normal cell activities such as cellular assembly and proliferation and mechanical properties of tissues [98]. To simulate this fibrous structure of ECM, nanofibrous scaffolds have increasingly been developed in recent years. Several techniques for fabricating nanofibers have been developed such as electrospinning, self-assembly, phase separation, and template synthesis. Among them, electrospinning has been regarded as an efficient and established technique capable of producing nanofibers by electrically charging a suspended droplet of polymer melt or solution. With this technique, a single polymer of synthetic or natural origin can be fabricated to nanofibrous scaffolds and, also, so can composite polymers.

Marine biomaterials have been fabricated to nanofibrous matrices by many researchers, and explored for various tissue engineering applications such as bone, cartilage, and skin tissue regeneration. Alginate is one of the great candidates for preparing nanofibrous matrices for tissue engineering. Jeong *et al.* demonstrated the promise of the electrospun alginate nanofibers with defined nanoscale architecture and cell adhesive properties for tissue regeneration applications [14]. They covalently coupled alginate with peptides having a cell adhesive sequence as alginate itself is not adhesive to cells. When preparing the alginate nanofibers, they blended alginate with PEO as the marine biopolymer cannot be electrospun alone due to a lack of chain entanglements [99]. Human dermal fibroblasts seeded within the alginate nanofiber modified with the RGD sequences exhibited spreading on the nanofiber, indicating the cells could adhere to the scaffolds well while few cells associated with the unmodified alginate nanofibers showed a round shape and did not attach to or spread on the nanofibers. In addition, a live/dead staining revealed the cells seeded on the modified alginate nanofibers extensively proliferated, and almost all the cells were viable.

Alginate has also been fabricated to core-shell nanofibrous matrices for tissue engineering applications by Ma *et al.* [15]. Core-shell nanofibers are promising in terms of their capability of incorporating drugs or bioactive molecules within the fibers [100]. This feature of core-shell nanofibers can be used to control cell behaviors more predictably. TEM images they obtained showed that alginate nanofibers were properly wrapped by the shell material PEO. Fibroblasts seeded within the core-shell alginate/PEO nanofibers attached on the nanofiber membranes and showed elongated and spindle-like morphology. The reason for this result may be attributed to the short-inter-nanofiber distance and high surface density of the nanofibers, which facilitate cell adhesion and spreading across the neighbor nanofibers [101]. The cells also could retain their viability in the nanofibrous scaffolds, indicating non-cytotoxicity of the core-shell nanofibers. Combining the biocompatibility of alginate and ECM-simulating physical structure of nanofibers, alginate-based nanofibers thus showed the great potential for tissue engineering applications.

##### Multilayer Microcapsules

During cell cultivation processes for cell therapy, therapeutics cells have not often maintained their viability and function. Even if the cells were successfully engineered to tissues, the tissues could be readily damaged during transplantation procedures. In addition, it is a great challenge to properly control the cells implanted inside the body. Thus, there has increasingly been need for effective strategies to overcome such hurdles.

One of the most promising approaches for addressing these problems is to exploit cell delivery systems or scaffolds composed of biopolymers having similarities with the natural ECM. As described above, ECM contains various proteins including the adhesion proteins and small bioactive molecules. ECM also influences all normal cell activities such as movement, development, repair, and regeneration. The existence of ECM is thus extremely crucial for cells.

Although numerous cell delivery systems and scaffolds have demonstrated improved outcomes, they were often fabricated in conditions intolerable to therapeutic cells, and could not efficiently accommodate cells during preparation or cell seeding procedures. Moreover, due to their voluminous nature, conventional cell delivery systems or scaffolds were not properly applied in some situations. In particular, when applying the biomaterial-based cell delivery systems or scaffolds to clinical practice, such defects could be more problematic.

In this context, multilayer microcapsule strategies have increasingly received interest as an alternative for conventional platforms for cell delivery and tissue engineering. Using the multilayer microcapsule approach, therapeutic cells can be safely encapsulated by very thin biomaterial-based envelopes simulating properties of natural ECM such as physical strength, viscoelasticity, porosity, and bioactive cues. In addition, the volume of cells multilayered with the biopolymer envelopes is much lower than that of common hydrogels and microparticular systems incorporating cells, thereby providing a smooth diffusive environment for gases and nutrients and greater possibility for application to clinical practice.

Alginate has been employed in multilayer microcapsule strategies due to its good biocompatibility and capability to form multilayers with other polymeric materials exhibiting positive charges under aqueous conditions. Miura *et al.* reported a novel approach to microencapsulate islets with a layer-by-layer technique using sodium alginate and poly(l-lysine) [18]. They at first modified the surface property of each cell using poly(ethylene glycol)-phospholipid (PEG-lipid) conjugates for protein anchoring. The PEG chains oriented towards outside of the cells exhibited positive charges. They added a solution of sodium alginate, exhibiting negative charges, to the PEG-lipid-modified islet suspension to form first layer on the surface of the islets. The islets were then exposed to a solution of poly(l-lysine) exhibiting positive charges to establish a second layer. These procedures were repeated to make multilayers composed of the ionic polymeric materials on the surface of the islets. Multilayer microcapsules were found to be successfully formed without a decrease in cell viability and significant increase in volume. The glucose stimulation test also confirmed that the insulin secreting ability of the islets was preserved after the multi-layering process. Other than this study, multilayer microcapsule approaches using alginate have been investigated for various applications such as transplantation device, immune protection, and stem cell therapy [102,103]. The significance of the multilayer microcapsule approaches for cell therapy applications would likely be greater in the future due to their unique advantages such as provision of minimally voluminal property, smooth, diffusive environments for cells, and capability to accurately recreate cell niche.

### 2.2. Chitosan

**Figure 3:**
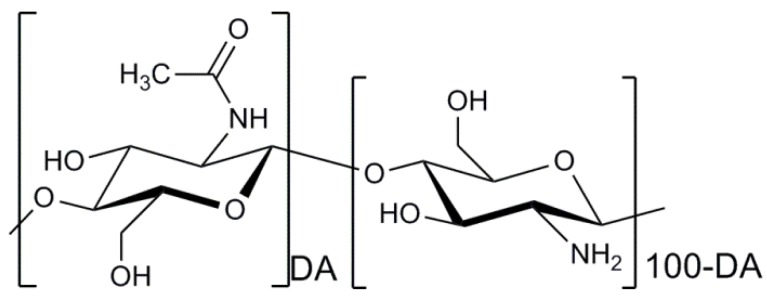
Chemical structure of chitosan. DA indicates the degree of *N*-acetylation of chitosan.

Chitosan is a copolymer of β-(1→4)-linked 2-acetamido-2-deoxy-d-glucopyranose and 2-amino-2-deoxy-d-glucopyranose as presented in Figure 3. The marine polysaccharide is not extensively present in nature, but can be obtained by deacetylation of the naturally occurring chitin extracted from the exoskeleton of marine organisms, mainly crabs and shrimps [104]. A distinguishing difference between chitin and chitosan is that the latter can form polyelectrolyte complexes due to its high cationicity (at least deacetylation degree of 60%) [105,106,107]. The presence of amino groups of the d-glucosamine residues in the chemical structure of chitosan generated by the deacetylation process makes chitosan positively charged in diluted acidic aqueous solutions (pH<6) [108]. It is a helpful property because the mechanical properties of chitosan-based cell delivery systems can be improved by the polyelectrolyte complexation under mild conditions to therapeutic cells. Indeed, chitosan is the only polysaccharide obtained from natural sources that can be positively charged [109]. Furthermore, the amino groups of d-glucosamine residues of chitosan can react with aldehyde groups of other molecules through reductive amination [110]. This reactivity of chitosan enables the marine polysaccharide to form stable covalent bonding with other molecules, particularly crosslinking agents such as genipin and glutaraldehyde used to modify its physicochemical properties [111].

Chitosan can also readily be fabricated to a variety of micromorphologies such as microspheres, fibers, and films from its acidic aqueous solutions [112]. The excellent ability to form porous structures simply by freezing and lyophilizing its solutions also makes chitosan a versatile biopolymer for tissue engineering, particularly in orthopedics for cartilage [113] and bone regeneration [114]. In addition to these advantages, chitosan possesses other noteworthy properties such as antibacterial activity [115,116], mucoadhesive [117], analgesic [118], and hemostatic properties [119]. The biodegradation rate of chitosan can also be simply controlled by varying its molecular weight and deacetylation degree [120,121]. The degradation products of chitosan are *N*-acetyl-glucosamine and glucosamine, natural constituents of the human body, and thus bioabsorbable [122,123]. Chitosan has shown good biocompatibility [124], and was approved by the FDA for use in wound dressings [125]. All these distinct advantages together indicate chitosan is one of the most remarkable candidates for cell therapy applications. However, similar to other biomaterials, in most cases, the requirements for cell therapy applications cannot be fulfilled by chitosan alone. Therefore, multifaceted strategies are necessary to maximize the utility of chitosan as a key biomaterial for satisfactory outcomes of cell therapy.

#### 2.2.1. Strategies for Designing Chitosan-Based Systems for Cell Therapy Applications

##### Enhancement of Cell Adhesiveness Using RGD Peptides

Although chitosan possess good biocompatibility and biodegradability, it also lacks bioactive signals for cell attachment, growth, and differentiation. In order to overcome this drawback, chitosan needs to be conjugated with the cell binding peptides. For this reason, chitosan also has been conjugated with the RGD amino acid sequences. Tsai *et al.* investigated RGD-modified three dimensional crosslinked chitosan scaffolds for bone tissue engineering [12]. To fabricate the chitosan scaffolds in which the peptides are uniformly distributed, they used mixtures of unmodified chitosan and two chitosan derivatives: one containing photoreactive azido groups for UV crosslinking and the other conjugated with RGD peptides. The mesenchymal stem cells (MSCs) cultured in the RGD-conjugated chitosan scaffolds prepared displayed elongated and spread morphology indicating they attached and spread well in the scaffolds while few cells were observed to attach and spread on the unmodified chitosan scaffolds. Furthermore, the MSCs cultured in the RGD-conjugated chitosan scaffolds showed a higher proliferation rate and more improved osteogenic differentiation, confirmed by several early and late osteogenic markers such as alkaline phosphatase [126], Runx2 [127], and osteocalcin [128], than those cultured in the control scaffolds.

RGD-modified chitosan has also been exploited for application in bone tissue engineering [13]. Scaffolds for bone tissue engineering were prepared using chitosan and hydroxyapatite (HA), a naturally occurring mineral form preferred for the stimulation of bone ingrowth, compositely. RGD peptides were then physically adsorbed to the scaffolds by immersing the scaffolds in RGD solutions. The bone marrow stromal cells were found to be adhered to the RGD-modified chitosan/HA scaffolds well, exhibiting a cell adhesion rate of around 80% for 4 h. This result indicates the RGD modification enhanced the cell affinity to the scaffolds. From a Live/Dead cell assay, it was observed that the cells were entirely viable and evenly distributed on the scaffolds, indicating the high cytocompatibility of the RGD-modified scaffolds. The cells also displayed a flattened polygonal shape and pseudopodia, and formed ECM. The elevated levels of alkaline phosphatase activity were confirmed from the cells on the RGD-modified chitosan/HA scaffolds, meaning the cells differentiated toward an osteogenic phenotype. From rabbit models with radial bone defects, the formation of bone tissue after eight weeks’ implantation of the RGD-modified scaffolds was observed under an X-ray tomography. These studies together indicate the RGD modification enhances the cell–scaffold interactions and promotes cell adhesion to scaffolds, as well as proliferation, and differentiation.

##### Optimization of Mechanical Characteristics

Although chitosan is a potential biomaterial for various cell therapy applications owing to its advantages such as biocompatibility, biodegradability, biological activities, and facile manipulation to diverse morphologies, one of the main drawbacks of chitosan-based cell delivery systems or scaffolds is their weak mechanical properties [129]. Chemical modifications have attracted great attention as a tool for enhancing mechanical properties of chitosan-based systems for cell therapy. In general, the chemical modification approaches have been carried out utilizing numerous amino and hydroxyl side groups chitosan processes. By varying experimental settings, the mechanical properties of the chitosan-based systems could be appropriately regulated. However, some reagents used to perform chemical modifications of chitosan have been reported to exhibit cytotoxicity [130], and the outcomes were sometimes not successful [131]. Thus, alternative strategies for reinforcing mechanical properties of the chitosan-based systems have been required.

Recently, chitosan fibers have been explored to enhance mechanical properties of the chitosan-based systems for cell therapy applications. Chitosan fibers with regular molecular arrangement and high orientation degree usually possess excellent breakdown strength and breakdown extension ratio [132]. Thus, mechanical properties of cell delivery systems or scaffolds can be reinforced by incorporating chitosan fibers. For instance, mechanical properties of tubular chitosan scaffolds for intestinal tissue engineering applications have been enhanced by circumferentially aligning chitosan fibers around the scaffolds by Zakhem *et al.* [133]. They prepared tubular chitosan scaffolds with inner fibers and/or outer fibers using a mold technique. As a result, the chitosan scaffolds incorporating chitosan fibers exhibited enhanced mechanical properties such as tensile strength, Young’s modulus, elongation at break, and burst pressure strength. Each value of the mechanical property parameters were significantly low in the case of tubular chitosan scaffolds without chitosan fibers exhibited compared to those of the native intestine. In contrast, the chitosan scaffolds on which chitosan fibers were circumferentially aligned showed similar mechanical strength to the native intestine. Smooth muscle cells aligned along the chitosan scaffolds with chitosan fibers successfully formed smooth muscle sheets. The α-smooth muscle actin was also positively stained from the cells, indicating preservation of smooth muscle phenotype.

In another study, variables affecting mechanical properties of chitosan fibers were investigated by Albanna *et al.* [134]. They characterized the effects of acetic acid concentration in the chitosan solution, pH of the coagulation bath, and annealing temperature on mechanical properties and crystallinity of chitosan fibers prepared. When 2% acetic acid was used to prepare chitosan solutions, the diameter of chitosan fibers fabricated was reduced and the mechanical properties were greatly enhanced. The reason for this may be attributed to the complete dissolving of the chitosan powders within the solution because a complete dissolving leads to the formation of chitosan fibers with a compact and dominant crystalline structure. However, the excess amount of acetic acid causes the increase in hydrogen bonding and resulting loosely-packed and extended structure of the chitosan fibers. Increasing the pH of the coagulation bath also resulted in compact and crystalline structure of chitosan fibers, reinforcing their mechanical properties. When adjusting the annealing temperature to around the glass transition temperature of chitosan, the mechanical strength of chitosan fibers prepared was enhanced as the crystallization of chitosan occurred dominantly at the temperature condition. The cell attachment and viability study demonstrated that the treatments for increasing mechanical strength of chitosan fiber scaffolds did not affect their cell-compatibility properties. Thus, chitosan fibers prepared under appropriate conditions can be used to reinforce intrinsic weak mechanical properties of chitosan-based systems for cell therapy applications.

##### Control of Porous Structures

The existence of porous properties in biodegradable cell delivery systems or scaffolds is extremely important because it facilitates the diffusion of nutrients and gases inside and outside cells incorporated in the matrix [135] and affects the cell ingrowth and seeding efficiency in the biodegradable substrate [136]. For this reason, efficient strategies for establishing porous structure in chitosan-based cell delivery systems or scaffolds are needed. In the case of chitosan, freeze-drying has been utilized as one of the most simple and effective methods for the purpose of producing such porous chitosan-based systems [137]. During the freeze-drying procedure, phase separation within chitosan solution occurs due to the difference between solubility of chitosan in the frozen and unfrozen region. After a complete sublimation of the solvent, pores are produced in the space in which the solvent existed prior to the sublimation stage.

For instance, Fang *et al.* prepared porous chitosan-based microspheres for cartilage regeneration using a freeze-drying method [19]. The porosity and average pore sizes could be increased by increasing the freezing temperature. This may be because the mass transfer rate is different at each temperature and the phase separation phenomenon occurred differently [138]. At −20 °C, the chitosan microspheres exhibited an average pore diameter of around 47 μm, which is in optimal range of pore diameter for cell seeding and growth in microcarriers [139,140]. *In vitro* study, mouse chondrocytes were found to be seeded and infiltrated well within the chitosan microspheres. The cells could retain their viability without significant decrease and proliferated successfully showing significantly increased cell numbers seven and 14 days after seeding. Freeze-drying can thus be usefully harnessed as a porous structure-establishing method for cell therapy applications.

However, the restrictive porous microstructure made by freeze-drying approaches is sometimes not ideal for cell therapy applications such as tissue engineering [141,142,143]. Other than the freeze-drying method, although various methods for preparing porous biodegradable systems for cell therapy applications have been developed such as gas forming [144], phase separation [145], and 3D printing [146], they are not appropriate to be applied to acidic chitosan solutions [20]. As usable alternatives to establish porous structure in chitosan-based systems for cell therapy applications, solvent casting and particle leaching methods have extensively been utilized. In general solvent casting and some leaching processes, a polymer is dissolved in an organic solvent and particles, typically salts, with specific sizes and shapes, are then added to the solution. The solution containing salts are usually placed in a mold to be shaped into final geometry. After evaporating the solvent, salts within the hardened polymer matrix are dissolved out, leaving behind a porous structure. The main advantage of this method is that porosity and pore size can be easily controlled by polymer/salt ratio and size of salt particles [147]. However, if the sizes and shapes of particles leached from biodegradable matrices are not uniform, it is difficult to obtain predictable pore sizes and shapes, which is undesirable for reproducible outcomes of cell seeding, infiltration, and tissue regeneration.

In the case of chitosan, a salt with a low ionization degree under a diluted acidic solution can only be used for establishing porous structure as the solvent for dissolving the polysaccharide is water. For instance, there is a study where sodium acetate with a relatively low pK_a_ (acetic acid = 4.76) was used as a porogen for preparing porous chitosan scaffolds [20]. The chitosan scaffolds fabricated showed uniform pore structure and good interconnectivity up to 90%. The porosity and interconnectivity could be controlled by varying the ratio of sodium acetate added. Cell adhesion on the porous chitosan scaffolds was observed, and the cells could proliferate successfully. In addition, the chitosan scaffolds with porous structure exhibited good mechanical properties in terms of mechanical strength, elongation at break, and elasticity.

As such, the solvent casting and particle leaching method can be applied to establish porous structure in chitosan-based systems. Nonetheless, this approach has also drawbacks such as its time-consuming procedure and limited controllability of pore sizes and shape. Therefore, there is still a need to develop strategies to fabricate porous chitosan systems with more simple procedures and controllable porous properties.

#### 2.2.2. Stimuli-Responsive Chitosan Systems for Cell Therapy Applications

##### Thermo-Responsive Chitosan Systems

As introduced in Section 2.1.2, thermo-responsive biomaterial-based systems are usefully exploited to fabricate injectable hydrogels and cell culturing platforms. Owing to the advantages of chitosan as a biomaterial for cell therapy applications, the marine biomaterial has been used to prepare thermo-responsive systems.

Wang *et al.* developed the thermo-responsive hydrogels using copolymerized polymers composed of acrylic acid-derivatized chitosan (CSA) and NIPAAm (poly(NIPAAm-co-CSA) [67]. The hydrogels composed of the poly(NIPAAm-co-CSA) were designed to achieve enhanced cell attachment and growth and a much more rapid cell sheet detachment for facile harvest of cultured cells. As a result, the degree of cell adhesion and spreading of the cultured model cells was greater on the copolymerized hydrogels than that obtained from PNIPAAm hydrogels without chitosan. The poly(NIPAAm-co-CSA) hydrogels exhibited much more cells adhering to the substrate when evaluated by a hemocytometer, SEM observation, and MTT assay. Furthermore, on increasing temperature from 20 °C to 37 °C, the cultured cells were more rapidly detached from the poly(NIPAAm-co-CSA) hydrogels than the PNIPAAm hydrogels because the hydrophilic surface property of CSA weakened cellular adhesion to the substrate. Furthermore, even when detached from the hydrogels, the cells were still alive and demonstrated properties similar to the attached condition.

Thermo-sensitive chitosan can also be exploited to fabricate injectable cell delivery systems [28]. Chen *et al.* prepared chitosan-graft-PNIPAAm hydrogels for application in cartilage tissue generation. They synthesized the copolymer by conjugating the carboxylic acid group of PNIPAAm to the amine group of chitosan, and produced the thermo-sensitive hydrogels with comb-like structure using the copolymer. The hydrogels prepared exhibited a gel formation temperature and a gel melting temperature of 29.8 °C and 28.3 °C, respectively. The gel formation was complete within 3 min. This fast gel formation rate would be beneficial for uniform cell distribution within the hydrogels. In addition, this property is desirable for injectable hydrogel applications. They seeded rabbit articular chondrocytes within the hydrogels and evaluated their biocompatibility and cell proliferation using a Live/Dead assay kit and an MTS assay. As a result, no significant difference in cell survival rate was observed between the chitosan-graft-PNIPAAm hydrogels and a tissue culture polystyrene dish controls. The reason for this result might be the structural similarity of chitosan to glycosaminoglycan, a major component of the ECM of chondrocytes, provided appropriate biological signals to the cells seeded within the hydrogels. When observed by SEM, the chondrocytes were embedded within secreted ECM and exhibited their phenotypes of a rounded cell shape, indicating they could maintain their functions and differentiation state. Combined together, the thermo-responsive chitosan-based systems are useful for cell cultivation and rapid tissue harvest without having undesirable effects on the tissues engineered, and can be used as a potential injectable hydrogel for cell therapy applications.

##### Electrically Responsive Chitosan Systems

Electroactive biomaterials are one of the newly developed stimuli-responsive biomaterials allowing electrical or electrochemical stimulation to cells incorporated in the biomaterial-based systems [148,149]. Among a variety of electroactive biomaterials, conductive polymers have demonstrated excellent control of the electrical stimulus, good electrical and optical properties, high conductivity/weight ratio, biocompatibility, and biodegradability [148,150,151]. In addition, the chemical, electrical, and physical characteristics of conductive polymers can be adjusted to meet the requirements of specific applications [148,152,153]. Considering these novel properties of conductive polymers, they have infinite possibilities of revolutionizing the cell therapy field.

For example, conductive polymers can be used to control cell functions by electrically stimulating cells, particularly electrically excitable cells including neuronal or muscle cells [154,155]. Furthermore, a myriad of studies have shown that neurite outgrowth and cell spreading can be significantly enhanced by electrically stimulating the cells through conductive polymers [154,156]. As a conductive polymer for such applications, polypyrrole (PPy) has recently been investigated for cell therapy applications due to their good stability and high conductance [157]. However, conductive polymers alone cannot fulfill the requisites for cell therapy applications such as microenvironments supporting cell adhesion, spreading, proliferation, and differentiation. In this context, chitosan and conductive polymers have been compositely used to prepare electrically responsive biomaterial-based systems in the cell therapy field.

As an instance, chitosan/PPy membranes have been investigated as a cell culture system for Schwann cells by Huang *et al.* [158]. They prepared the chitosan/PPy membranes by physically mixing chitosan (97.5%) and PPy (2.5%) in a diluted acidic solution, followed by drying the mixture. After culturing Schwann cells on the membrane prepared to 95% confluence, they applied a lateral constant potential gradient (100 mV/mm, 4 h) to the Schwann cells through the chitosan/PPy membrane using a custom-built electrical cell culture system. As a result, the chitosan/PPy membrane promoted cell adhesion, spreading, and proliferation with or without electrical stimulation. When stimulating the cells electrically, their viability was significantly increased, which was confirmed by SEM observation, DAPI staining, and MTT assay. Furthermore, the electrical stimulation through the membrane enhanced the expression and secretion of nerve growth factor and brain-derived neurotrophic factor, evaluated by RT-PCR assay and Western blot analysis, in comparison to control cells without the electrical stimulation.

In another study, aniline pentamer (AP) crosslinking chitosan (AP-c-CS) was used to fabricate electrically responsive scaffolds for neural tissue regeneration [159]. Aniline pentamer (AP) was used as a conductive material due to its good electroactivity and biodegradability [160]. The AP-c-CS was synthesized by a condensation polymerization technique. The scaffolds prepared with the AP-c-CS, and rat neuronal pheochromocytoma PC-12 cells were seeded in the scaffolds. The cells seeded in the AP-c-CS scaffolds showed better cell adhesion and proliferation rates than pure AP or CS scaffolds due to the combined effect of the cytocompatibility of chitosan and electrically active AP. The AP-c-CS scaffolds also promoted the neuronal differentiation largely due to their electrical stimulation. During the differentiation process, the morphology of PC-12 cells gradually changed from the circular shape to the neuronal phenotype. However, in the case of scaffolds fabricated with pure CS, few cells produced neurites, indicating the natural biomaterial alone could not promote the cells to differentiate. Thus, the combinatorial utilization of advantages of marine biomaterials such as chitosan and conductive polymers are promising for cell therapy applications including neural tissue engineering.

#### 2.2.3. Platforms for Cell Delivery and Tissue Engineering

##### Hydrogels

As described in Section 2.1.3, hydrogels are one of the most appropriate formulations for cell therapy applications due to their unique advantages. Like alginate, chitosan has also been extensively explored as a basic material for preparing hydrogels for cell delivery and tissue engineering purposes. Chitosan hydrogels have demonstrated outstanding biocompatibility, enzyme-mediated biodegradability, and non-toxicity even from their degradation products.

Although chitosan hydrogels have such advantages, they do not lead to successful outcomes due to their shortcomings such as lack of control over its gelation time, insufficiency of biological activity, inability to tune their mechanical properties, and solubility tissues at physiological pH under specific situations. Many researchers have devised a variety of strategies to overcome such problems.

For example, in the case of chitosan hydrogels fabricated by chemical crosslinking methods, cytotoxicity of crosslinking agents may be problematic. Thus, therapeutic cells are needed to be incorporated in the chitosan hydrogels after preparation process. However, in this case, shearing forces for distributing the cells within the hydrogels can reduce the cell viability and function. Lack of ability to fill empty spaces generated by an injury is also a disadvantage of chemically crosslinked chitosan hydrogels. In this context, photocrosslinkable chitosan-based hydrogels have been developed. As an instance, Valmikinathan *et al.* synthesized photocrosslinkable chitosan modified with methacrylate for neural tissue engineering [161]. Using the methacrylate chitosan, hydrogels were prepared under UV exposure for 3–5 min. The chitosan hydrogels were formed 3 min after UV radiation, demonstrating *in situ* gelation capability. The hydrogels also showed controllable rheological properties with varying chitosan concentration, demonstrating the formation of a dense network due to high availability of the methacrylate groups on chitosan. When human MSCs were incubated within the chitosan hydrogels, no significant changes in the cell viability and morphology were detected, and the cell number was similar to that of the cells incubated in tissue culture plate wells examined as a control. In addition, the cells cultured in the photo-crosslinked chitosan hydrogels showed significantly enhanced neurite outgrowth, while an agarose-based hydrogel did not lead to the neuron growth to the same degree observed from the chitosan hydrogel.

To enhance biological activities of chitosan, bioactive factors or natural polymers having bioactive properties have been applied to chitosan hydrogels. These materials can stimulate various cell activities including cell growth, proliferation, function, and differentiation. For instance, collagen and transforming growth factor-beta (TGF-β) have been applied to chitosan hydrogels to facilitate cartilage tissue engineering by enhancing chondrogenic differentiation by Kim *et al.* [162,163,164]. In the study, TGF-β-conjugated chitosan and collagen were fabricated into hydrogels using a photopolymerization method. Due to the presence of TGF-β and collagen within chitosan hydrogels, MSCs derived from human synovium proliferated better than hydrogels without the bioactive materials. *In vitro* chondrogenic differentiation of the cells was also examined by histology (H & E staining and Safranin-O) and immunohistochemistry (collagen staining). The encapsulated cells exhibited round shape with surrounding lacunae, indicating the chondrogenic differentiation. In addition, increased accumulation of glycosaminoglycan and expression of collagen were detected from the cells cultured on the collagen-impregnated TGF-β-conjugated chitosan-based hydrogels. The cells also showed increased expression level of chondrogenic gene markers such as Sox 9, aggrecan, and type II collagen.

Besides these approaches, there are numerous possible strategies for maximizing the potential of chitosan hydrogels for cell therapy applications such as peptide modification [165] and application of thermo-sensitive polymers to render the hydrogels injectable [166]. The utility of chitosan hydrogels as cell delivery systems and scaffolds can be increased by using novel strategies that will be devised in the future.

##### Nanofibrous Matrices

Along with alginate, chitosan is one of the most extensively explored marine biomaterials to fabricate nanofibrous matrices for cell therapy applications. Although chitosan is not appropriate for preparing nanofibers using electrospinning techniques due to the viscous nature of its solution and strong hydrogen bonds formed within the polymer network precluding the movement of polymeric chains under the electrical field exposure [167,168], this challenge has been overcome by blending chitosan with synthetic polymers including polyvinyl alcohol (PVA) and PEO [169]. Chitosan-based nanofibers have shown remarkable outcomes in various tissue engineering applications due to the combined advantages of chitosan and nanofibers as aforementioned above.

For instance, chitosan-based nanofibrous matrices have been explored for skin tissue regeneration. Chitosan is a promising biomaterial for skin tissue regeneration purposes because it activates fibroblast proliferation and promotes collagen deposition by secreting *N*-acetyl-β-glucosamine [170,171]. In addition, the polysaccharide stimulates the wound healing process by inducing the high level of hyaluronic acid at the wound location [172]. Nanofibrous matrices are also a great candidate as a wound dressing material because their porous structure is advantageous for cell seeding and diffusion of nutrients, oxygen, and waste [173], and they can absorb wound exudates, preventing excessive dehydration and bacterial infection from the wound [173,174]. Furthermore, nanofibrous matrices prepared from biomaterials have been known to activate the fibroblasts excreting the main constituents of ECM to heal the injured tissue [175].

In a study, chitosan nanofibrous matrices were fabricated using an electrospinning technique [16]. Their effect on morphology, proliferation, and differentiation of skin tissue-related cells (fibroblasts, keratinocytes, and endothelial cells) were compared with films and sponges prepared with chitosan, followed by wound healing assay using a mouse model. When observing the cells seeded after culture period, the sponges showed several drawbacks such as poor cell attachment, impossibility of gaining monolayers, and low number of cells seeded. As for the chitosan films, the cells incubated failed to spread fully, and stopped multiplying rapidly. However, the cells seeded on the electrospun chitosan nanofibrous matrices fully spread on the substrates, and proliferated well with time, thereby forming cell clusters of flat cells tightly joined together. In addition, the keratinocytes seeded on the scaffolds differentiated successfully showing the expression of differentiation markers such as keratin 14 and involucrin. The chitosan nanofibrous matrices also demonstrated its excellent biocompatibility exhibiting the recovery of full thickness wounds *in vivo* study.

Chitosan nanofibrous matrices have also attracted immense attention in the bone tissue engineering field. Chitosan itself is promising for bone tissue engineering, mainly due to its biocompatibility and structural resemblance to bone ECM [176,177]. The nanofibrous architecture may also be advantageous for bone tissue engineering as it promotes the proliferation, differentiation, and mineralization of osteoprogenitor cells [178]. Incorporation of bioactive substances promoting bone ingrowth within chitosan nanofibers may maximize the potential of the nanofibrous matrices’ ability to regenerate bone tissues [179,180]. Frohbergh *et al.* investigated this approach for bone tissue engineering [17]. They prepared HA-containing chitosan nanofibrous matrices crosslinked with genipin. The HA-incorporating chitosan nanofibrous matrices were prepared by electrospinning a chitosan solution where HA nanoparticles were dispersed in advance. The crosslinking of chitosan using genipin rendered the resulting chitosan nanofibrous matrices more similar to natural bones in terms of mechanical properties. It is noteworthy to highlight that the chitosan nanofibrous matrices were fabricated without the use of a fiber-forming agent such as PEO. By controlling various conditions such as the humidity, ambient temperature, and the concentration and degree of deacetylation of chitosan, they could produce chitosan nanofibrous matrices using only chitosan and HA as components of the fibers. At 7 days post-seeding, osteoblasts showed the well-defined filopodia extending from the lamellipodia, indicating the cells could interact with the chitosan nanofibrous matrices. Furthermore, the cells formed confluent monolayers and a rough texture on the nanofibrous scaffold, implying the continued proliferation and an enhanced maturation of osteoblasts. The cells also exhibited high expression of alkaline phosphatase, an indicator of osteogenic differentiation [181].

Due to the promising features of chitosan nanofibrous matrices, they have also been utilized for other tissue engineering purposes including cardiac tissue regeneration [182]. In the future, many more strategies will be devised for increasing the utility of chitosan nanofibrous matrices for tissue engineering applications, and, as such, their range of applications will broaden.

##### Multilayer Microcapsules

As introduced in Section 2.1.3, multilayer microcapsules composed of ionic polymers are very promising platforms for a variety of cell therapy applications due to their ability to provide ECM-mimicking microenvironments for cells and their minimal voluminal nature. This strategy has also been usefully applied to cellular therapeutics releasing biological molecules in a sustained manner [183], and to prevent immune rejections of hosts against therapeutic cells derived from allogeneic sources [21]. To fully utilize the multilayer microcapsule approach, selection of biocompatible polymers with appropriate ionic properties is critical. Polymers used to envelope therapeutic cells must be cytocompatible, biodegradable, and able to be processed in mild conditions. In addition, the polymers should not cause loss of cell function, and in some cases, must not stimulate cells to release their biological molecules too early. From this perspective, chitosan has been regarded to be an attractive biomaterial for use in the multilayer microcapsule approach. Although only few research reports on the multilayer microcapsule strategy are available, they have shown promise for cell therapy applications.

For instance, Zhi *et al.* used islets enveloped with multilayers composed of chitosan and sodium alginate [22]. They first added a chitosan solution to cells to initiate film growth on the cell surfaces, followed by adsorption of sodium alginate to the surface of chitosan thin film in the same manner to form a single chitosan/alginate bilayer. This process was repeated until a desired multilayer of (chitosan/alginate)*_n_* chitosan was formed, where “*n*” represents the number of bilayers. They monitored the multilayer deposition on cell surfaces by measuring zeta-potential, and the average thickness of each dried layer was determined to be about 1.5 nm. The cells enveloped with the marine biomaterials showed no sign of apoptosis, suggesting the cells were healthy and not affected by the multilayers. The insulin secreting ability of the islets also was not compromised. This result suggests that the marine biomaterial-based cell coating technique has potential for biological molecule-releasing cell therapy applications.

In another study, living platelets were encapsulated by a thin multilayer consisting of chitosan and poly-l-glutamic acid [21]. The platelets can be effectively used to repair blood vessels, and for clot formation, retraction, and dissolution due to biological actions induced by the release of their contents. However, the cells can lose their bio-functions too fast when administered to the body. In this context, the authors applied the multilayer microcapsule strategy in the platelet-based cell therapy as the encapsulation of the cells can lead to sustained release of the biological molecules such as growth factors, and can reduce immune rejection generating from hosts. The encapsulation of the platelets was performed by self-assembly of the polyelectrolytes using a layer-by-layer technology. As a result, the cell contents and structure were retained well before and after the multi-layering process. In contrast, the cells multilayered with ionic polymers different from chitosan and poly-l-glutamic acid were activated during the encapsulation process, thereby releasing their contents and losing their functions too early.

Although the multilayer microcapsule strategies have not been fully investigated, they would attract much more attention by researchers in the biomedical field in the future owing to their unique advantages such as immune protection, controlled release of biological factors produced by cells, capability to provide ECM-mimicking environments, and minimal voluminal nature.

### 2.3. Miscellaneous Marine Biomaterials

**Figure 4:**
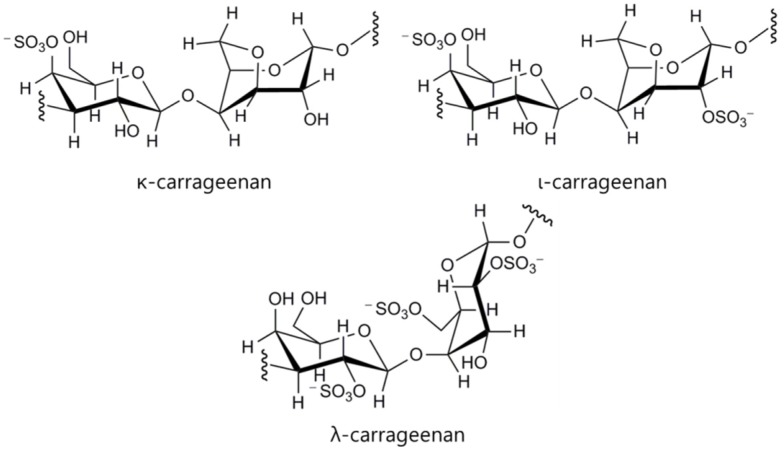
Chemical structure of kappa (κ), iota (ι), and lambda (λ)-carrageenans.

While alginate and chitosan are leading marine biomaterials investigated for cell therapy applications so far, other marine biomaterials also have recently shown great promise. Among them, carrageenan has most actively been explored for cell therapy purposes. Carrageenans are sulfated polysaccharides produced as a matrix material in several species of red seaweeds (Rhodophyceae) such as *Chondrus crispus*, *Gigartina*, and *Eucheuma cottonii* [184,185]. The polysaccharide can be categorized as three main families according to the number and position of sulfate group in the repeating galactose units: kappa (κ), iota (ι), and lambda (λ) as seen in Figure 4. Among them, κ-carrageenan has primarily been exploited in the cell therapy field because of its distinguishing properties. Hydrogels prepared with κ-carrageenan are thermo-reversible, and can be readily prepared by an ionic gelation mechanism: potassium cations contained within κ-carrageenan prevent the electrostatic repulsion between the neighboring helices exhibiting negative charges, thereby promoting their aggregation [186,187]. With the ionic gelation mechanism, cells can be incorporated within κ-carrageenan hydrogels in a mild condition. Its processability to various shape/formats and resemblance of chemical structure to glycosaminoglycans are distinctive advantages that can be exploited from κ-carrageenan [188]. The injectability of κ-carrageenan hydrogels under physiological conditions is also one of the appealing features of the marine biomaterial. Although this marina biomaterial has only recently been used in cell therapy applications, it has demonstrated promising performances in several studies.

For example, Santo *et al.* proposed carrageenan-based hydrogels capable of releasing platelet derived growth factor (PDGF) with controlled rates for bone tissue engineering applications [189]. They prepared κ-carrageenan beads using an ionotropic gelation method, followed by loading PDGF into the bead. The loading efficiency of PDGF in the beads could also be controlled by changing the processing parameters such as the biomaterial concentration, the ionic crosslinking agent, namely, potassium chloride, and the hardening time in the crosslinking medium, exhibiting up to around 90%. In addition, release behavior of PDGF from the beads could also be controlled depending on the processing parameters. From an MTS assay, fibroblasts incubated in extracts prepared with theκ-carrageenan beads did not exhibit significant reduction of their viability, indicating thatκ-carrageenan beads are cytocompatible. The authors suggested the κ-carrageenan beads are a promising injectable system that can be used to deliver cells and growth factors to the body in a minimally invasive manner.

Carrageenan has also demonstrated the potential in cartilage tissue engineering. The marine polysaccharide is specifically advantageous for cartilage tissue regeneration due to its structural similarity to glycosaminoglycans, one of the key components constituting the ECM of the cartilage tissue [190,191]. Rocha *et al.* reported carrageenan-based hydrogels encapsulating hADSCs and TGF-β1 to engineer cartilage tissue [192]. The reason for incorporation of TGF-β1 in the carrageenan-based hydrogels was because it can initiate cell–cell interactions and stimulate chondrocyte proliferation and differentiation [193,194,195] as well as production of proteoglycans and other components of cartilage matrix [196]. The carrageenan hydrogels incorporating TGF-β1 enhanced the cell viability and proliferation, and increased the expression level of chondrogenic differentiation markers, demonstrating the promise of κ-carrageenan for cartilage tissue engineering.

More recently, Popa *et al.* also reported on κ-carrageenan hydrogel with encapsulated hASCs for cartilage tissue engineering [197]. The κ-carrageenan hydrogels did not exhibit any cytotoxic effect on hASCs, corroborated with the fluorescence staining and DNA quantification experiments. The authors demonstrated the κ-carrageenan hydrogels supported the cells’ functionality and construction of ECM with detection of glycosaminoglycans’ deposition and proteoglycans’ protein production with a metachromatic staining technique. In addition, the mechanical analysis showed enhanced stiffness and viscoelastic properties of the κ-carrageenan gels with their encapsulated cells during the culture period. The chondrogenic differentiation was also confirmed with the expression of type II collagen by the cells. These studies suggest that carrageenan-based hydrogels offer novel approaches for the treatment of cartilage defects.

Carrageenan has also been compositely used with alginate for preparing hydrogel beads and fibers, and demonstrated good processability to different formulations for cell delivery and tissue engineering applications [198]. As shown in these literatures, carrageenan has distinguishable advantages in terms of biological properties and processability to diverse injectable hydrogel-based formulations. It is thus expected that carrageenan will be a leading marine biomaterial, following alginate and chitosan, for cell therapy applications.

**Figure 5:**
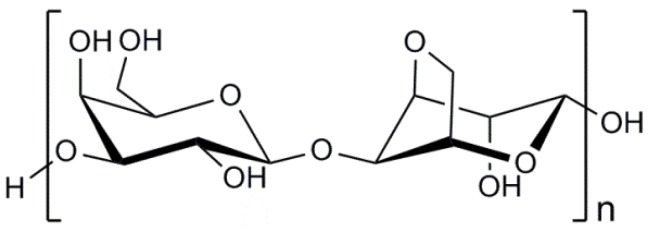
Chemical structure of agarose.

In addition to carrageenan, agarose (Figure 5) has also been explored for cell therapy applications. Agarose is a polysaccharide polymer material derived from certain species of red algae (*Gelidium*, *Gelidiela*, *Pterocladia*, *Gracilaria*, *Graciliaropsis*, and *Ahfeltia*) [199]. The marine polysaccharide is composed of linearly polymerized repeating units of arabinose, which is a disaccharide made up of d-galactose and 3,6-anhydro-l-galactopyranose [200]. A distinguishing property of agarose is its capability to form thermo-responsive gels transformed at around physiological temperatures [201]. Owing to the thermo-sensitive property, agarose hydrogels have been investigated for drug and cell delivery applications in the biomedical field [202,203,204]. In particular, the marine polysaccharide has been used to prepare scaffolds for engineering of soft tissues such as neural [205] and cartilage tissues [206,207] due to its soft tissue-like mechanical characteristics and biocompatibility. The mechanical properties of agarose-based hydrogels can also be controlled by gelling temperatures and curing times [208]. However, agarose has low cell adhesiveness and biological properties to promote cellular activities including proliferation and differentiation [207]. For this reason, agarose has generally been investigated with other materials having biological properties for cell therapy applications.

For instance, gelatin-conjugated agarose hydrogels have been used as a tissue engineering scaffold [209]. In the study, gelatin was used to give the agarose-based scaffolds cell-adhesive property. The cells attached and spread on the agarose-gelatin scaffolds well, while no cell adhesion was observed on the hydrogels composed only of agarose. The number of cells seeded on the agarose-gelatin hydrogels was not significantly different from that on tissue culture dishes. In another study, agarose was used along with chitosan and gelatin to prepare scaffolds for cartilage tissue engineering due to its good mechanical strength and capacity to retain chondrocytes phenotype [210]. The hydrogels prepared with the composite materials demonstrated appropriate mechanical and porous characteristics and successful outcomes in *in vivo* experiments. Agarose has also been fabricated to hydrogels with a porous structure [211], and used to prepare scaffolds together with cellulose [212] for tissue engineering applications, exhibiting excellent performances. This agarose is a potential marine polysaccharide as a basic material to prepare biocompatible systems for cell delivery and tissue engineering purposes, and its application ranges are expected to be broadened in the future.

Recently, exopolysaccharides (EPS) derived from micro-organisms inhabiting extreme marine environments have attracted growing interest from researchers in the cell therapy field [213]. The micro-organisms have unique metabolic pathways to survive in extreme environments, and secrete special biological materials such as EPS. The EPS derived from different marine micro-organisms display novel chemical compositions and unique biological activities. However, most of the EPS remain poorly understood, and only a few of them have been fully characterized. The promising biological properties of microbial EPS in the ocean identified so far include supporting cell adhesion, facilitating biochemical interactions between cells, providing protective action to cells, and absorbing dissolved organic materials needed to cells [214]. For example, EPS2, a polysaccharide secreted by a marine filamentous fungus *Keissleriella* sp., demonstrated remarkable free radical-scavenging actions [215,216]. This property can be utilized to mitigate the oxidative damage of therapeutic cells and enhance the cells’ natural defense ability [217,218]. EPS derived from marine fungus *Penicillium* sp. also exhibited considerable anti-oxidative activities, in particular scavenging abilities on superoxide radicals [219]. EPS obtained from *Vibrio diabolicus*, a bacterium isolated from a deep-sea hydrothermal vent polychaete annelid, were reported to be a strong bone-healing material as they promoted the bone restoration process in an experimental model [220]. EPS derived from fermentation of *Vibrio diabolicus* also exhibited a bone-regenerating activity when implanted to a mouse model having bone injuries [220]. As such, EPS produced by marine micro-organisms present novel biological activities that can be exploited for cell therapy applications. Although these polysaccharides have not actively been investigated yet as platform materials for cell therapy applications, due to their novel biological properties, they are expected to be increasingly employed by researchers in the cell therapy field.

## 3. Conclusions and Future Perspectives

Cell therapy, as a promising alternative for organ transplantation, has exhibited great potential for regenerating damaged or diseased tissues. To maximize therapeutic efficacies of the cell therapy, therapeutic cells were needed to be appropriately processed, delivered or implanted to target localities. Biomaterial-based platforms for cell delivery and tissue engineering scaffolds have played pivotal roles in meeting such requirements. As fundamental materials for fabricating such biomaterial-based platforms, marine biopolymers have been extensively investigated due to the diversity of their chemical and biological properties and better biocompatibility than synthetic polymers and biomaterials derived from other natural sources. However, the marine biomaterial-based platforms sometimes did not meet all the conditions required for successful cell delivery and tissue engineering. In this context, various strategies have been suggested to enhance their performance, and have led to improved outcomes. Although the marine biomaterial platforms heretofore have only demonstrated their performance in *in vitro* and *in vivo* models mainly with scientific indicators representing cell viability, proliferation rate, and differentiation so far, the evaluation of the platforms would be performed in terms of practical examination of functionality of regenerated tissues. Novel strategies that will be developed in the future would contribute largely to meeting advanced evaluation standards. Based on this, marine biomaterial-based platforms for cell therapy will sometimes be able to be applied in clinical practices.

## References

[1] Jana S., Tefft B.J., Spoon D.B., Simari R.D. (2014). Scaffolds for tissue engineering of cardiac valves. Acta Biomater..

[2] Zhang L., Hu J., Athanasiou K.A. (2011). The role of tissue engineering in articular cartilage repair and regeneration. Crit. Rev. Biomed. Eng..

[3] Bose S., Roy M., Bandyopadhyay A. (2012). Recent advances in bone tissue engineering scaffolds. Trends Biotechnol..

[4] Shah A., Brugnano J., Sun S., Vase A., Orwin E. (2008). The development of a tissue-engineered cornea: Biomaterials and culture methods. Pediatr. Res..

[5] Haraguchi Y., Shimizu T., Yamato M., Okano T. (2012). Concise review: Cell therapy and tissue engineering for cardiovascular disease. Stem Cells Transl. Med..

[6] Lee K.Y., Mooney D.J. (2012). Alginate: Properties and biomedical applications. Prog. Polym. Sci..

[7] Lee K.Y., Yuk S.H. (2007). Polymeric protein delivery systems. Prog. Polym. Sci..

[8] Pawar S.N., Edgar K.J. (2012). Alginate derivatization: A review of chemistry, properties and applications. Biomaterials.

[9] Tan H., Gong Y., Lao L., Mao Z., Gao C. (2007). Gelatin/chitosan/hyaluronan ternary complex scaffold containing basic fibroblast growth factor for cartilage tissue engineering. J. Mater. Sci. Mater. Med..

[10] Shachar M., Tsur-Gang O., Dvir T., Leor J., Cohen S. (2011). The effect of immobilized RGD peptide in alginate scaffolds on cardiac tissue engineering. Acta Biomater..

[11] Sandvig I., Karstensen K., Rokstad A.M., Aachmann F.L., Formo K., Sandvig A., Skjåk-Bræk G., Strand B.L. (2014). RGD-peptide modified alginate by a chemoenzymatic strategy for tissue engineering applications. J. Biomed. Mater. Res. Part A.

[12] Tsai W.B., Chen Y.R., Li W.T., Lai J.Y., Liu H.L. (2012). RGD-conjugated UV-crosslinked chitosan scaffolds inoculated with mesenchymal stem cells for bone tissue engineering. Carbohydr. Polym..

[13] Chen L., Li B., Xiao X., Meng Q., Li W., Yu Q., Bi J., Cheng Y., Qu Z. (2015). Preparation and evaluation of an Arg-Gly-Asp-modified chitosan/hydroxyapatite scaffold for application in bone tissue engineering. Mol. Med. Rep..

[14] Jeong S.I., Krebs M.D., Bonino C.A., Khan S.A., Alsberg E. (2010). Electrospun alginate nanofibers with controlled cell adhesion for tissue engineering. Macromol. Biosci..

[15] Ma G., Fang D., Liu Y., Zhu X., Nie J. (2012). Electrospun sodium alginate/poly(ethylene oxide) core-shell nanofibers scaffolds potential for tissue engineering applications. Carbohydr. Polym..

[16] Tchemtchoua V.T., Atanasova G., Aqil A., Filée P., Garbacki N., Vanhooteghem O., Deroanne C., Noël A., Jérome C., Nusgens B. (2011). Development of a chitosan nanofibrillar scaffold for skin repair and regeneration. Biomacromolecules.

[17] Frohbergh M.E., Katsman A., Botta G.P., Lazarovici P., Schauer C.L., Wegst U.G.K., Lelkes P.I. (2012). Electrospun hydroxyapatite-containing chitosan nanofibers crosslinked with genipin for bone tissue engineering. Biomaterials.

[18] Miura S., Teramura Y., Iwata H. (2006). Encapsulation of islets with ultra-thin polyion complex membrane through poly(ethylene glycol)-phospholipids anchored to cell membrane. Biomaterials.

[19] Fang J., Zhang Y., Yan S., Liu Z., He S., Cui L., Yin J. (2014). Poly(l-glutamic acid)/chitosan polyelectrolyte complex porous microspheres as cell microcarriers for cartilage regeneration. Acta Biomater..

[20] Lim J.I., Lee Y.K., Shin J.S., Lim K.J. (2011). Preparation of interconnected porous chitosan scaffolds by sodium acetate particulate leaching. J. Biomater. Sci. Polym. Ed..

[21] Zhao Q., Li H., Li B. (2011). Nanoencapsulating living biological cells using electrostatic layer-by-layer self-assembly: Platelets as a model. J. Mater. Res..

[22] Zhi Z.L., Liu B., Jones P.M., Pickup J.C. (2010). Polysaccharide multilayer nanoencapsulation of insulin-producing β-cells grown as pseudoislets for potential cellular delivery of insulin. Biomacromolecules.

[23] Kong H.J., Lee K.Y., Mooney D.J. (2002). Decoupling the dependence of rheological/mechanical properties of hydrogels from solids concentration. Polymer.

[24] Lee K.Y., Bouhadir K.H., Mooney D.J. (2000). Degradation behavior of covalently cross-linked poly(aldehyde guluronate) hydrogels. Macromolecules.

[25] Kong H.J., Kaigler D., Kim K., Mooney D.J. (2004). Controlling rigidity and degradation of alginate hydrogels via molecular weight distribution. Biomacromolecules.

[26] Kong H.J., Alsberg E., Kaigler D., Lee K.Y., Mooney D.J. (2004). Controlling degradation of hydrogels via the size of cross-linked junctions. Adv. Mater..

[27] Abdi S.I.H., Choi J.Y., Lee J.S., Lim H.J., Lee C., Kim J., Chung H.Y., Lim J.O. (2012). *In vivo* study of a blended hydrogel composed of pluronic F-127-alginate-hyaluronic acid for its cell injection application. Tissue Eng. Regen. Med..

[28] Chen J.P., Cheng T.H. (2006). Thermo-responsive chitosan-graft-poly(*N*-isopropylacrylamide) injectable hydrogel for cultivation of chondrocytes and meniscus cells. Macromol. Biosci..

[29] Tan R., She Z., Wang M., Fang Z., Liu Y., Feng Q. (2012). Thermo-sensitive alginate-based injectable hydrogel for tissue engineering. Carbohydr. Polym..

[30] Zhao X., Kim J., Cezar C.A., Huebsch N., Lee K., Bouhadir K., Mooney D.J. (2011). Active scaffolds for on-demand drug and cell delivery. Proc. Natl. Acad. Sci. USA.

[31] Cezar C.A., Kennedy S.M., Mehta M., Weaver J.C., Gu L., Vandenburgh H., Mooney D.J. (2014). Biphasic ferrogels for triggered drug and cell delivery. Adv. Healthc. Mater..

[32] Zhao L.B., Pan L., Zhang K., Guo S.S., Liu W., Wang Y., Chen Y., Zhao X.Z., Chan H.L.W. (2009). Generation of Janus alginate hydrogel particles with magnetic anisotropy for cell encapsulation. Lab Chip.

[33] Montjovent M.O., Mark S., Mathieu L., Scaletta C., Scherberich A., Delabarde C., Zambelli P.Y., Bourban P.E., Applegate L.A., Pioletti D.P. (2008). Human fetal bone cells associated with ceramic reinforced PLA scaffolds for tissue engineering. Bone.

[34] Cao H., Kuboyama N. (2010). A biodegradable porous composite scaffold of PGA/beta-TCP for bone tissue engineering. Bone.

[35] Pan Z., Ding J. (2012). Poly (lactide-co-glycolide) porous scaffolds for tissue engineering and regenerative medicine. Interface Focus.

[36] Sung H.J., Meredith C., Johnson C., Galis Z.S. (2004). The effect of scaffold degradation rate on three-dimensional cell growth and angiogenesis. Biomaterials.

[37] Senni K., Pereira J., Gueniche F., Delbarre-Ladrat C., Sinquin C., Ratiskol J., Godeau G., Fischer A.M., Helley D., Colliec-Jouault S. (2011). Marine polysaccharides: A source of bioactive molecules for cell therapy and tissue engineering. Mar. Drugs.

[38] Sun J., Tan H. (2013). Alginate-based biomaterials for regenerative medicine applications. Materials.

[39] Smidsrød O., Skjåk-Bræk G. (1990). Alginate as immobilization matrix for cells. Trends Biotechnol..

[40] Tønnesen H.H., Karlsen J. (2002). Alginate in drug delivery systems. Drug Dev. Ind. Pharm..

[41] George M., Abraham T.E. (2006). Polyionic hydrocolloids for the intestinal delivery of protein drugs: Alginate and chitosan—A review. J. Control. Release.

[42] Draget K.I., Skjåk-Bræk G., Stokke B.T. (2006). Similarities and differences between alginic acid gels and ionically crosslinked alginate gels. Food Hydrocoll..

[43] Bouhadir K.H., Lee K.Y., Alsberg E., Damm K.L., Anderson K.W., Mooney D.J. (2001). Degradation of partially oxidized alginate and its potential application for tissue engineering. Biotechnol. Prog..

[44] Balakrishnan B., Jayakrishnan A. (2005). Self-cross-linking biopolymers as injectable *in situ* forming biodegradable scaffolds. Biomaterials.

[45] Lee K.Y., Kong H.J., Larson R.G., Mooney D.J. (2003). Hydrogel formation via cell crosslinking. Adv. Mater..

[46] Lehenkari P.P., Horton M.A. (1999). Single integrin molecule adhesion forces in intact cells measured by atomic force microscopy. Biochem. Biophys. Res. Commun..

[47] Koo L.Y., Irvine D.J., Mayes A.M., Lauffenburger D.A., Griffith L.G. (2002). Co-regulation of cell adhesion by nanoscale RGD organization and mechanical stimulus. J. Cell Sci..

[48] Hersel U., Dahmen C., Kessler H. (2003). RGD modified polymers: Biomaterials for stimulated cell adhesion and beyond. Biomaterials.

[49] Ruoslahti E. (1996). RGD and other recognition sequences for integrins. Annu. Rev. Cell Dev. Biol..

[50] Michel J.B. (2003). Anoikis in the cardiovascular system: Known and unknown extracellular mediators. Arterioscler. Thromb. Vasc. Biol..

[51] Valentijn A.J., Zouq N., Gilmore A.P. (2004). Anoikis. Biochem. Soc. Trans..

[52] Kuo C.K., Ma P.X. (2001). Ionically crosslinked alginate hydrogels as scaffolds for tissue engineering: Part 1. Structure, gelation rate and mechanical properties. Biomaterials.

[53] Ruvinov E., Leor J., Cohen S. (2010). The effects of controlled HGF delivery from an affinity-binding alginate biomaterial on angiogenesis and blood perfusion in a hindlimb ischemia model. Biomaterials.

[54] Man Y., Wang P., Guo Y., Xiang L., Yang Y., Qu Y., Gong P., Deng L. (2012). Angiogenic and osteogenic potential of platelet-rich plasma and adipose-derived stem cell laden alginate microspheres. Biomaterials.

[55] Lee C.S.D., Watkins E., Burnsed O.A., Schwartz Z., Boyan B.D. (2013). Tailoring adipose stem cell trophic factor production with differentiation medium components to regenerate chondral defects. Tissue Eng. Part A.

[56] Gantenbein-Ritter B., Chan S.C.W. (2012). The evolutionary importance of cell ratio between notochordal and nucleus pulposus cells: An experimental 3-D co-culture study. Eur. Spine J..

[57] Jing W., Lin Y., Wu L., Li X., Nie X., Liu L., Tang W., Zheng X., Tian W. (2007). Ectopic adipogenesis of preconditioned adipose-derived stromal cells in an alginate system. Cell Tissue Res..

[58] Oliveira S.M., Barrias C.C., Almeida I.F., Costa P.C., Ferreira M.R.P., Bahia M.F., Barbosa M.A. (2008). Injectability of a bone filler system based on hydroxyapatite microspheres and a vehicle with *in situ* gel-forming ability. J. Biomed. Mater. Res. B. Appl. Biomater..

[59] Fonseca K.B., Bidarra S.J., Oliveira M.J., Granja P.L., Barrias C.C. (2011). Molecularly designed alginate hydrogels susceptible to local proteolysis as three-dimensional cellular microenvironments. Acta Biomater..

[60] Chang S.C.N., Chung H.Y., Tai C.L., Chen P.K.T., Lin T.M., Jeng L.B. (2010). Repair of large cranial defects by hBMP-2 expressing bone marrow stromal cells: Comparison between alginate and collagen type I systems. J. Biomed. Mater. Res. A.

[61] Kang S.W., Cha B.H., Park H., Park K.S., Lee K.Y., Lee S.H. (2011). The effect of conjugating RGD into 3D alginate hydrogels on adipogenic differentiation of human adipose-derived stromal cells. Macromol. Biosci..

[62] LeRoux M.A., Guilak F., Setton L.A. (1999). Compressive and shear properties of alginate gel: Effects of sodium ions and alginate concentration. J. Biomed. Mater. Res..

[63] Kong H.J., Smith M.K., Mooney D.J. (2003). Designing alginate hydrogels to maintain viability of immobilized cells. Biomaterials.

[64] Smidsrød O., Glover R.M., Whittington S.G. (1973). The relative extension of alginates having different chemical composition. Carbohydr. Res..

[65] Al-Shamkhani A., Duncan R. (1995). Radioiodination of alginate via covalently-bound tyrosinamide allows monitoring of its fate *in vivo*. J. Bioact. Compat. Polym..

[66] Tan H., Marra K.G. (2010). Injectable, biodegradable hydrogels for tissue engineering applications. Materials.

[67] Wang J., Chen L., Zhao Y., Guo G., Zhang R. (2009). Cell adhesion and accelerated detachment on the surface of temperature-sensitive chitosan and poly(*N*-isopropylacrylamide) hydrogels. J. Mater. Sci. Mater. Med..

[68] Mata J.P., Majhi P.R., Guo C., Liu H.Z., Bahadur P. (2005). Concentration, temperature, and salt-induced micellization of a triblock copolymer Pluronic L64 in aqueous media. J. Colloid Interface Sci..

[69] Fusco S. (2006). Perspectives on: PEO-PPO-PEO triblock copolymers and their biomedical applications. J. Bioact. Compat. Polym..

[70] He C., Kim S.W., Lee D.S. (2008). *In situ* gelling stimuli-sensitive block copolymer hydrogels for drug delivery. J. Control. Release.

[71] Cao Y., Rodriguez A., Vacanti M., Ibarra C., Arevalo C., Vacanti C.A. (1998). Comparative study of the use of poly(glycolic acid), calcium alginate and pluronics in the engineering of autologous porcine cartilage. J. Biomater. Sci. Polym. Ed..

[72] Sosnik A., Sefton M.V. (2005). Semi-synthetic collagen/poloxamine matrices for tissue engineering applications. Biomaterials.

[73] Bromberg L. (1998). Properties of aqueous solutions and gels of poly(ethylene oxide)-b-poly(propylene oxdie)-b-poly(ethylene oxide)-g-poly(acrylic acid). J. Phys. Chem. B.

[74] Kabanov A.V., Alakhov V.Y. (2002). Pluronic block copolymers in drug delivery: From micellar nanocontainers to biological response modifiers. Crit. Rev. Ther. Drug Carrier Syst..

[75] Zhong C., Wu J., Reinhart-King C.A., Chu C.C. (2010). Synthesis, characterization and cytotoxicity of photo-crosslinked maleic chitosan-polyethylene glycol diacrylate hybrid hydrogels. Acta Biomater..

[76] Tran N.Q., Joung Y.K., Lih E., Park K.M., Park K.D. (2010). Supramolecular hydrogels exhibiting fast *in situ* gel forming and adjustable degradation properties. Biomacromolecules.

[77] Goa K.L., Benfield P. (1994). Hyaluronic acid. A review of its pharmacology and use as a surgical aid in ophthalmology, and its therapeutic potential in joint disease and wound healing. Drugs.

[78] Drury J.L., Mooney D.J. (2003). Hydrogels for tissue engineering: Scaffold design variables and applications. Biomaterials.

[79] Zrínyi M., Barsi L., Büki A. (1996). Deformation of ferrogels induced by nonuniform magnetic fields. J. Chem. Phys..

[80] Zrínyi M., Barsi L., Büki A. (1997). Ferrogel: A new magneto-controlled elastic medium. Polym. Gels Netw..

[81] Dainiak M.B., Kumar A., Galaev I.Y., Mattiasson B. (2006). Detachment of affinity-captured bioparticles by elastic deformation of a macroporous hydrogel. Proc. Natl. Acad. Sci. USA.

[82] Yu Z., Xiaoliang W., Xuman W., Hong X., Hongchen G. (2008). Acute toxicity and irritation of water-based dextran-coated magnetic fluid injected in mice. J. Biomed. Mater. Res. A.

[83] Huebsch N., Arany P.R., Mao A.S., Shvartsman D., Ali O.A., Bencherif S.A., Rivera-Feliciano J., Mooney D.J. (2010). Harnessing traction-mediated manipulation of the cell/matrix interface to control stem-cell fate. Nat. Mater..

[84] Guilak F., Cohen D.M., Estes B.T., Gimble J.M., Liedtke W., Chen C.S. (2009). Control of stem cell fate by physical interactions with the extracellular matrix. Cell Stem Cell.

[85] Dendukuri D., Hatton T.A., Doyle P.S. (2007). Synthesis and self-assembly of amphiphilic polymeric microparticles. Langmuir.

[86] Nisisako T., Torii T. (2008). Microfluidic large-scale integration on a chip for mass production of monodisperse droplets and particles. Lab Chip.

[87] Grieshaber S.E., Jha A.K., Farran A.J.E., Jia X. (2011). Hydrogels in tissue engineering. Biomaterials for Tissue Engineering Applications.

[88] Morais J.M., Papadimitrakopoulos F., Burgess D.J. (2010). Biomaterials/tissue interactions: Possible solutions to overcome foreign body response. AAPS J..

[89] Bidarra S.J., Barrias C.C., Granja P.L. (2014). Injectable alginate hydrogels for cell delivery in tissue engineering. Acta Biomater..

[90] Salgado C.L., Sanchez E.M., Zavaglia C.A., Almeida A.B., Granja P.L. (2012). Injectable biodegradable polycaprolactone-sebacic acid gels for bone tissue engineering. Tissue Eng. Part A.

[91] Fonseca K.B., Maia F.R., Cruz F.A., Andrade D., Juliano M.A., Granja P.L., Barrias C.C. (2013). Enzymatic, physicochemical and biological properties of MMP-sensitive alginate hydrogels. Soft Matter.

[92] Munarin F., Guerreiro S.G., Grellier M.A., Tanzi M.C., Barbosa M.A., Petrini P., Granja P.L. (2011). Pectin-based injectable biomaterials for bone tissue engineering. Biomacromolecules.

[93] Bidarra S.J., Barrias C.C., Fonseca K.B., Barbosa M.A., Soares R.A., Granja P.L. (2011). Injectable *in situ* crosslinkable RGD-modified alginate matrix for endothelial cells delivery. Biomaterials.

[94] Kim W.S., Mooney D.J., Arany P.R., Lee K., Huebsch N., Kim J. (2012). Adipose tissue engineering using injectable, oxidized alginate hydrogels. Tissue Eng. Part A.

[95] Thornton A.J., Alsberg E., Hill E.E., Mooney D.J. (2004). Shape retaining injectable hydrogels for minimally invasive bulking. J. Urol..

[96] Wang L., Shansky J., Borselli C., Mooney D.J., Vandenburgh H. (2012). Design and fabrication of a biodegradable, covalently crosslinked shape-memory alginate scaffold for cell and growth factor delivery. Tissue Eng. Part A.

[97] Lee K.Y., Rowley J.A., Eiselt P., Moy E.M., Bouhadir K.H., Mooney D.J. (2000). Controlling mechanical and swelling properties of alginate hydrogels independently by cross-linker type and cross-linking density. Macromolecules.

[98] Sahay R., Kumar P.S., Sridhar R., Sundaramurthy J., Venugopal J., Mhaisalkar S.G., Ramakrishna S. (2012). Electrospun composite nanofibers and their multifaceted applications. J. Mater. Chem..

[99] Nie H., He A., Zheng J., Xu S., Li J., Han C.C. (2008). Effects of chain conformation and entanglement on the electrospinning of pure alginate. Biomacromolecules.

[100] Elahi F., Lu W., Guoping G., Khan F. (2013). Core-shell fibers for biomedical applications—A review. Bioeng. Biomed. Sci. J..

[101] Duan B., Yuan X., Zhu Y., Zhang Y., Li X., Zhang Y., Yao K. (2006). A nanofibrous composite membrane of PLGA-chitosan/PVA prepared by electrospinning. Eur. Polym. J..

[102] Leung A., Ramaswamy Y., Munro P., Lawrie G., Nielsen L., Trau M. (2005). Emulsion strategies in the microencapsulation of cells: Pathways to thin coherent membranes. Biotechnol. Bioeng..

[103] Karoubi G., Ormiston M.L., Stewart D.J., Courtman D.W. (2009). Single-cell hydrogel encapsulation for enhanced survival of human marrow stromal cells. Biomaterials.

[104] Burrows F., Louime C., Abazinge M., Onokpise O. (2007). Extraction and evaluation of chitosan from crab exoskeleton as a seed fungicide and plant growth enhancer. Am. Eurasian J. Agric. Environ. Sci..

[105] Acosta N., Jiménez C., Borau V., Heras A. (1993). Extraction and characterization of chitin from crustaceans. Biomass Bioenergy.

[106] Madihally S.V., Matthew H.W.T. (1999). Porous chitosan scaffolds for tissue engineering. Biomaterials.

[107] Muzzarelli R., Mehtedi M., Mattioli-Belmonte M. (2014). Emerging biomedical applications of nano-chitins and nano-chitosans obtained via advanced eco-friendly technologies from marine resources. Mar. Drugs.

[108] Leedy M., Martin H., Norowski P.A., Jennings A., Haggard W., Bumgardner J., Jennings J.A., Haggard W., Bumgardner J. (2011). Use of chitosan as a bioactive implant coating for bone-implant applications. Chitosan for Biomaterials II SE.

[109] Pavinatto F.J., Caseli L., Oliveira O.N. (2010). Chitosan in nanostructured thin films. Biomacromolecules.

[110] Rinaudo M. (2006). Chitin and chitosan: Properties and applications. Prog. Polym. Sci..

[111] Lai J.Y. (2012). Biocompatibility of genipin and glutaraldehyde cross-linked chitosan materials in the anterior chamber of the eye. Int. J. Mol. Sci..

[112] Tuzlakoglu K., Alves C.M., Mano J.F., Reis R.L. (2004). Production and characterization of chitosan fibers and 3-D fiber mesh scaffolds for tissue engineering applications. Macromol. Biosci..

[113] Suh J.K., Matthew H.W. (2000). Application of chitosan-based polysaccharide biomaterials in cartilage tissue engineering: A review. Biomaterials.

[114] Di Martino A., Sittinger M., Risbud M.V. (2005). Chitosan: A versatile biopolymer for orthopaedic tissue-engineering. Biomaterials.

[115] Sudarshan N.R., Hoover D.G., Knorr D. (1992). Antibacterial action of chitosan. Food Biotechnol..

[116] Ong S.Y., Wu J., Moochhala S.M., Tan M.H., Lu J. (2008). Development of a chitosan-based wound dressing with improved hemostatic and antimicrobial properties. Biomaterials.

[117] Lehr C.M., Bouwstra J.A., Schacht E.H., Junginger H.E. (1992). *In vitro* evaluation of mucoadhesive properties of chitosan and some other natural polymers. Int. J. Pharm..

[118] Aranaz I., Mengíbar M., Harris R., Paños I., Miralles B., Acosta N., Galed G., Heras Á. (2009). Functional characterization of chitin and chitosan. Curr. Chem. Biol..

[119] Yang J., Tian F., Wang Z., Wang Q., Zeng Y.J., Chen S.Q. (2008). Effect of chitosan molecular weight and deacetylation degree on hemostasis. J. Biomed. Mater. Res. B. Appl. Biomater..

[120] Vandevord P.J., Matthew H.W.T., Desilva S.P., Mayton L., Wu B., Wooley P.H. (2002). Evaluation of the biocompatibility of a chitosan scaffold in mice. J. Biomed. Mater. Res..

[121] Sashiwa H., Aiba S.I. (2004). Chemically modified chitin and chitosan as biomaterials. Prog. Polym. Sci..

[122] Bagheri-Khoulenjani S., Taghizadeh S.M., Mirzadeh H. (2009). An investigation on the short-term biodegradability of chitosan with various molecular weights and degrees of deacetylation. Carbohydr. Polym..

[123] Vårum K.M., Myhr M.M., Hjerde R.J.N., Smidsrød O. (1997). *In vitro* degradation rates of partially *N*-acetylated chitosans in human serum. Carbohydr. Res..

[124] Thanou M., Verhoef J.C., Junginger H.E. (2001). Oral drug absorption enhancement by chitosan and its derivatives. Adv. Drug. Deliv. Rev..

[125] Wedmore I., McManus J.G., Pusateri A.E., Holcomb J.B. (2006). A special report on the chitosan-based hemostatic dressing: Experience in current combat operations. J. Trauma.

[126] Grunkemeier J.M., Tsai W.B., Alexander M.R., Castner D.G., Horbett T.A. (2000). Platelet adhesion and procoagulant activity induced by contact with radiofrequency glow discharge polymers: Roles of adsorbed fibrinogen and vWF. J. Biomed. Mater. Res..

[127] Otto F., Thornell A.P., Crompton T., Denzel A., Gilmour K.C., Rosewell I.R., Stamp G.W., Beddington R.S., Mundlos S., Olsen B.R. (1997). Cbfa1, a candidate gene for cleidocranial dysplasia syndrome, is essential for osteoblast differentiation and bone development. Cell.

[128] Makita N., Suzuki M., Asami S., Takahata R., Kohzaki D., Kobayashi S., Hakamazuka T., Hozumi N. (2008). Two of four alternatively spliced isoforms of RUNX2 control osteocalcin gene expression in human osteoblast cells. Gene.

[129] Albanna M.Z., Bou-Akl T.H., Walters H.L., Matthew H.W.T. (2012). Improving the mechanical properties of chitosan-based heart valve scaffolds using chitosan fibers. J. Mech. Behav. Biomed. Mater..

[130] Mi F.L., Tan Y.C., Liang H.C., Huang R.N., Sung H.W. (2001). *In vitro* evaluation of a chitosan membrane cross-linked with genipin. J. Biomater. Sci. Polym. Ed..

[131] Hirano S., Nagamura K., Zhang M., Kim S.K., Chung B.G., Yoshikawa M., Midorikawa T. (1999). Chitosan staple fibers and their chemical modification with some aldehydes. Carbohydr. Polym..

[132] Wang X., Song G., Lou T., Peng W. (2009). Fabrication of nano-fibrous PLLA scaffold reinforced with chitosan fibers. J. Biomater. Sci. Polym. Ed..

[133] Zakhem E., Bitar K.N. (2015). Development of chitosan scaffolds with enhanced mechanical properties for intestinal tissue engineering applications. J. Funct. Biomater..

[134] Albanna M.Z., Bou-Akl T.H., Blowytsky O., Walters H.L., Matthew H.W.T. (2013). Chitosan fibers with improved biological and mechanical properties for tissue engineering applications. J. Mech. Behav. Biomed. Mater..

[135] Khademhosseini A., Langer R. (2007). Microengineered hydrogels for tissue engineering. Biomaterials.

[136] Torigoe I., Sotome S., Tsuchiya A., Yoshii T., Takahashi M., Kawabata S., Shinomiya K. (2007). Novel cell seeding system into a porous scaffold using a modified low-pressure method to enhance cell seeding efficiency and bone formation. Cell Transplant..

[137] Garg T., Chanana A., Joshi R. (2012). Preparation of chitosan scaffolds for tissue engineering using freeze drying technology. IOSR J. Pharm..

[138] Adriana Martel-Estrada S., Alberto Martinez-Perez C., Guadalupe Chacon-Nava J., Elvia Garcia-Casillas P., Olivas-Armendariz I. (2010). Synthesis and thermo-physical properties of chitosan/poly(dl-lactide-co-glycolide) composites prepared by thermally induced phase separation. Carbohydr. Polym..

[139] Huang C.C., Wei H.J., Yeh Y.C., Wang J.J., Lin W.W., Lee T.Y., Hwang S.M., Choi S.W., Xia Y., Chang Y. (2012). Injectable PLGA porous beads cellularized by hAFSCs for cellular cardiomyoplasty. Biomaterials.

[140] Malda J., Frondoza C.G. (2006). Microcarriers in the engineering of cartilage and bone. Trends Biotechnol..

[141] Adekogbe I., Ghanem A. (2005). Fabrication and characterization of DTBP-crosslinked chitosan scaffolds for skin tissue engineering. Biomaterials.

[142] Shirosaki Y., Okayama T., Tsuru K., Hayakawa S., Osaka A. (2008). Synthesis and cytocompatibility of porous chitosan-silicate hybrids for tissue engineering scaffold application. Chem. Eng. J..

[143] Hsieh W.C., Chang C.P., Lin S.M. (2007). Morphology and characterization of 3D micro-porous structured chitosan scaffolds for tissue engineering. Colloids Surf. B Biointerfaces.

[144] Murphy W.L., Kohn D.H., Mooney D.J. (2000). Growth of continuous bonelike mineral within porous poly(lactide-co-glycolide) scaffolds *in vitro*. J. Biomed. Mater. Res..

[145] Hua F.J., Park T.G., Lee D.S. (2003). A facile preparation of highly interconnected macroporous poly(d,l-lactic acid-co-glycolic acid) (PLGA) scaffolds by liquid-liquid phase separation of a PLGA-dioxane-water ternary system. Polymer.

[146] Sherwood J.K., Riley S.L., Palazzolo R., Brown S.C., Monkhouse D.C., Coates M., Griffith L.G., Landeen L.K., Ratcliffe A. (2002). A three-dimensional osteochondral composite scaffold for articular cartilage repair. Biomaterials.

[147] Liu C. (2207). Design and development of three-dimensional scaffolds for tissue engineering. Chem. Eng. Res. Des..

[148] Lakard B., Ploux L., Anselme K., Lallemand F., Lakard S., Nardin M., Hihn J.Y. (2009). Effect of ultrasounds on the electrochemical synthesis of polypyrrole, application to the adhesion and growth of biological cells. Bioelectrochemistry.

[149] Ghasemi-Mobarakeh L., Prabhakaran M.P., Morshed M., Nasr-Esfahani M.H., Baharvand H., Kiani S., Al-Deyab S.S., Ramakrishna S. (2011). Application of conductive polymers, scaffolds and electrical stimulation for nerve tissue engineering. J. Tissue Eng. Regen. Med..

[150] Kotwal A., Schmidt C.E. (2001). Electrical stimulation alters protein adsorption and nerve cell interactions with electrically conducting biomaterials. Biomaterials.

[151] Lee J.Y., Bashur C.A., Goldstein A.S., Schmidt C.E. (2009). Polypyrrole-coated electrospun PLGA nanofibers for neural tissue applications. Biomaterials.

[152] Wallace G.G., Smyth M., Zhao H. (1999). Conducting electroactive polymer-based biosensors. Trends Anal. Chem..

[153] Kim D.H., Richardson-Burns S.M., Hendricks J.L., Sequera C., Martin D.C. (2007). Effect of immobilized nerve growth factor on conductive polymers: Electrical properties and cellular response. Adv. Funct. Mater..

[154] Schmidt C.E., Shastri V.R., Vacanti J.P., Langer R. (1997). Stimulation of neurite outgrowth using an electrically conducting polymer. Proc. Natl. Acad. Sci. USA.

[155] Nishizawa M., Nozaki H., Kaji H., Kitazume T., Kobayashi N., Ishibashi T., Abe T. (2007). Electrodeposition of anchored polypyrrole film on microelectrodes and stimulation of cultured cardiac myocytes. Biomaterials.

[156] Zhang Z., Rouabhia M., Wang Z., Roberge C., Shi G., Roche P., Li J., Dao L.H. (2007). Electrically conductive biodegradable polymer composite for nerve regeneration: Electricity-stimulated neurite outgrowth and axon regeneration. Artif. Organs.

[157] Guimard N.K., Gomez N., Schmidt C.E. (2007). Conducting polymers in biomedical engineering. Prog. Polym. Sci..

[158] Huang J., Hu X., Lu L., Ye Z., Zhang Q., Luo Z. (2010). Electrical regulation of Schwann cells using conductive polypyrrole/chitosan polymers. J. Biomed. Mater. Res. A.

[159] Hu J., Huang L., Zhuang X., Zhang P., Lang L., Chen X., Wei Y., Jing X. (2008). Electroactive aniline pentamer cross-linking chitosan for stimulation growth of electrically sensitive cells. Biomacromolecules.

[160] Huang L., Hu J., Lang L., Wang X., Zhang P., Jing X., Wang X., Chen X., Lelkes P.I., MacDiarmid A.G. (2007). Synthesis and characterization of electroactive and biodegradable ABA block copolymer of polylactide and aniline pentamer. Biomaterials.

[161] Valmikinathan C.M., Mukhatyar V.J., Jain A., Karumbaiah L., Dasari M., Bellamkonda R.V. (2012). Photocrosslinkable chitosan based hydrogels for neural tissue engineering. Soft Matter.

[162] Kim J., Lin B., Kim S., Choi B., Evseenko D., Lee M. (2015). TGF-β1 conjugated chitosan collagen hydrogels induce chondrogenic differentiation of human synovium-derived stem cells. J. Biol. Eng..

[163] Ha C.W., Noh M.J., Choi K.B., Lee K.H. (2012). Initial phase I safety of retrovirally transduced human chondrocytes expressing transforming growth factor-beta-1 in degenerative arthritis patients. Cytotherapy.

[164] Zhang L., Yuan T., Guo L., Zhang X. (2012). An *in vitro* study of collagen hydrogel to induce the chondrogenic differentiation of mesenchymal stem cells. J. Biomed. Mater. Res. A.

[165] Park K.M., Lee S.Y., Joung Y.K., Na J.S., Lee M.C., Park K.D. (2009). Thermosensitive chitosan—Pluronic hydrogel as an injectable cell delivery carrier for cartilage regeneration. Acta Biomater..

[166] Reis L.A., Chiu L.L., Liang Y., Hyunh K., Momen A., Radisic M. (2012). A peptide-modified chitosan-collagen hydrogel for cardiac cell culture and delivery. Acta Biomater..

[167] Geng X., Kwon O.H., Jang J. (2005). Electrospinning of chitosan dissolved in concentrated acetic acid solution. Biomaterials.

[168] Neamnark A., Rujiravanit R., Supaphol P. (2006). Electrospinning of hexanoyl chitosan. Carbohydr. Polym..

[169] Duan B., Dong C., Yuan X., Yao K. (2004). Electrospinning of chitosan solutions in acetic acid with poly(ethylene oxide). J. Biomater. Sci. Polym. Ed..

[170] Howling G.I., Dettmar P.W., Goddard P.A., Hampson F.C., Dornish M., Wood E.J. (2001). The effect of chitin and chitosan on the proliferation of human skin fibroblasts and keratinocytes *in vitro*. Biomaterials.

[171] Dai T., Tanaka M., Huang Y.Y., Hamblin M.R. (2011). Chitosan preparations for wounds and burns: Antimicrobial and wound-healing effects. Expert Rev. Anti Infect. Ther..

[172] Paul W., Sharma C. (2004). Chitosan and alginate wound dressings: A short review. Trends Biomater. Artif. Organs.

[173] Khil M.S., Cha D.I., Kim H.Y., Kim I.S., Bhattarai N. (2003). Electrospun nanofibrous polyurethane membrane as wound dressing. J. Biomed. Mater. Res. B Appl. Biomater..

[174] Muzzarelli R.A. (2011). Biomedical exploitation of chitin and chitosan via mechano-chemical disassembly, electrospinning, dissolution in imidazolium ionic liquids, and supercritical drying. Mar. Drugs.

[175] Chen J.P., Chang G.Y., Chen J.K. (2008). Electrospun collagen/chitosan nanofibrous membrane as wound dressing. Colloids Surf. A Physicochem. Eng. Asp..

[176] Zhang Y., Venugopal J.R., El-Turki A., Ramakrishna S., Su B., Lim C.T. (2008). Electrospun biomimetic nanocomposite nanofibers of hydroxyapatite/chitosan for bone tissue engineering. Biomaterials.

[177] Kumirska J., Czerwicka M., Kaczyński Z., Bychowska A. (2010). Application of spectroscopic methods for structural analysis of chitin and chitosan. Mar. Drugs.

[178] Woo K.M., Jun J.H., Chen V.J., Seo J., Baek J.H., Ryoo H.M., Kim G.S., Somerman M.J., Ma P.X. (2007). Nano-fibrous scaffolding promotes osteoblast differentiation and biomineralization. Biomaterials.

[179] Ito Y., Hasuda H., Kamitakahara M., Ohtsuki C., Tanihara M., Kang I.K., Kwon O.H. (2005). A composite of hydroxyapatite with electrospun biodegradable nanofibers as a tissue engineering material. J. Biosci. Bioeng..

[180] Kim H.W., Lee H.H., Knowles J.C. (2006). Electrospinning biomedical nanocomposite fibers of hydroxyapatite/poly(lactic acid) for bone regeneration. J. Biomed. Mater. Res. Part A.

[181] Saad F.A., Hofstaetter J.G. (2011). Proteomic analysis of mineralising osteoblasts identifies novel genes related to bone matrix mineralisation. Int. Orthop..

[182] Hussain A., Collins G., Yip D., Cho C.H. (2013). Functional 3-D cardiac co-culture model using bioactive chitosan nanofiber scaffolds. Biotechnol. Bioeng..

[183] Krol S., del Guerra S., Grupillo M., Diaspro A., Gliozzi A., Marchetti P. (2006). Multilayer nanoencapsulation. new approach for immune protection of human pancreatic islets. Nano Lett..

[184] Pereira L., Amado A.M., Critchley A.T., van de Velde F., Ribeiro-Claro P.J. (2009). Identification of selected seaweed polysaccharides (phycocolloids) by vibrational spectroscopy (FTIR-ATR and FT-Raman). Food Hydrocoll..

[185] Rivera-Carro H., Craigie J.S., Shacklock P.F. (1990). Influence of tissue source and growth rates on dry weight and carrageenan composition of *Chondrus crispus* (Gigartinales, Rhodophyta). Hydrobiologia.

[186] Wang Q., Rademacher B., Sedlmeyer F., Kulozik U. (2005). Gelation behaviour of aqueous solutions of different types of carrageenan investigated by low-intensity-ultrasound measurements and comparison to rheological measurements. Innov. Food Sci. Emerg. Technol..

[187] Kara S., Arda E., Pekcan Ö. (2007). Monovalent and divalent ction effects on phase transitions of ι-carrageenan. J. Bioact. Compat. Polym..

[188] Popa E.G., Carvalho P.P., Dias A.F., Santos T.C., Santo V.E., Marques A.P., Viegas C.A., Dias I.R., Gomes M.E., Reis R.L. (2014). Evaluation of the *in vitro* and *in vivo* biocompatibility of carrageenan-based hydrogels. J. Biomed. Mater. Res..

[189] Santo V.E., Frias A.M., Carida M., Cancedda R., Gomes M.E., Mano J.F., Reis R.L. (2009). Carrageenan-based hydrogels for the controlled delivery of PDGF-BB in bone tissue engineering applications. Biomacromolecules.

[190] Bhattacharyya S., Liu H., Zhang Z., Jam M., Dudeja P.K., Michel G., Linhardt R.J., Tobacman J.K. (2010). Carrageenan-induced innate immune response is modified by enzymes that hydrolyze distinct galactosidic bonds. J. Nutr. Biochem..

[191] Silva T.H., Alves A., Popa E.G., Reys L.L., Gomes M.E., Sousa R.A., Silva S.S., Mano J.F., Reis R.L. (2012). Marine algae sulfated polysaccharides for tissue engineering and drug delivery approaches. Biomatter.

[192] Rocha P.M., Santo V.E., Gomes M.E., Reis R.L., Mano J.F. (2011). Encapsulation of adipose-derived stem cells and transforming growth factor-Î^2^1 in carrageenan-based hydrogels for cartilage tissue engineering. J. Bioact. Compat. Polym..

[193] Holland T.A., Mikos A.G. (2003). Advances in drug delivery for articular cartilage. J. Control. Release.

[194] Tuli R., Tuli S., Nandi S., Huang X., Manner P.A., Hozack W.J., Danielson K.G., Hall D.J., Tuan R.S. (2003). Transforming growth factor-beta-mediated chondrogenesis of human mesenchymal progenitor cells involves *N*-cadherin and mitogen-activated protein kinase and Wnt signaling cross-talk. J. Biol. Chem..

[195] Betre H., Ong S.R., Guilak F., Chilkoti A., Fermor B., Setton L.A. (2006). Chondrocytic differentiation of human adipose-derived adult stem cells in elastin-like polypeptide. Biomaterials.

[196] Park H., Temenoff J.S., Holland T.A., Tabata Y., Mikos A.G. (2005). Delivery of TGF-beta1 and chondrocytes via injectable, biodegradable hydrogels for cartilage tissue engineering applications. Biomaterials.

[197] Popa E.G., Caridade S.G., Mano J.F., Reis R.L., Gomes M.E. (2015). Chondrogenic potential of injectable κ-carrageenan hydrogel with encapsulated adipose stem cells for cartilage tissue-engineering applications. J. Tissue Eng. Regen. Med..

[198] Popa E.G., Gomes M.E., Reis R.L. (2011). Cell delivery systems using alginate-carrageenan hydrogel beads and fibers for regenerative medicine applications. Biomacromolecules.

[199] Araki C. (1966). Some recent studies on the polysaccharides of agarophytes. Proc. Int. Seaweed Symp..

[200] Lee S.B., Cho S.J., Kim J.A., Lee S.Y., Kim S.M., Lim H.S. (2014). Metabolic pathway of 3,6-anhydro-l-galactose in agar-degrading microorganisms. Biotechnol. Bioprocess Eng..

[201] Normand V., Lootens D.L., Amici E., Plucknett K., Aymard P. (2000). New insight into agarose gel mechanical properties. Biomacromolecules.

[202] Liu J., Li L. (2005). SDS-aided immobilization and controlled release of camptothecin from agarose hydrogel. Eur. J. Pharm. Sci..

[203] Yang H., Iwata H., Shimizu H., Takagi T., Tsuji T., Ito F. (1994). Comparative studies of *in vitro* and *in vivo* function of three different shaped bioartificial pancreases made of agarose hydrogel. Biomaterials.

[204] Sakai S., Kawabata K., Ono T., Ijima H., Kawakami K. (2005). Development of mammalian cell-enclosing subsieve-size agarose capsules (<100 microm) for cell therapy. Biomaterials.

[205] Jain A., Kim Y.T., McKeon R.J., Bellamkonda R.V. (2006). *In situ* gelling hydrogels for conformal repair of spinal cord defects, and local delivery of BDNF after spinal cord injury. Biomaterials.

[206] Gruber H.E., Hoelscher G.L., Leslie K., Ingram J.A., Hanley E.N. (2006). Three-dimensional culture of human disc cells within agarose or a collagen sponge: Assessment of proteoglycan production. Biomaterials.

[207] Gruber H.E., Fisher E.C., Desai B., Stasky A.A., Hoelscher G., Hanley E.N. (1997). Human intervertebral disc cells from the annulus: Three-dimensional culture in agarose or alginate and responsiveness to TGF-beta1. Exp. Cell Res..

[208] Aymard P., Martin D., Plucknett K. (2001). Influence of thermal history on the structural and mechanical properties of agarose gels. Biopolymers.

[209] Sakai S., Hashimoto I., Kawakami K. (2007). Synthesis of an agarose-gelatin conjugate for use as a tissue engineering scaffold. J. Biosci. Bioeng..

[210] Bhat S., Tripahi A., Kumar A. (2011). Supermacroprous chitosan-agarose-gelatin cryogels: *In vitro* characterization and *in vivo* assessment for cartilage tissue engineering. J. R. Soc. Interface.

[211] Park J.H., Chung B.G., Lee W.G., Kim J., Brigham M.D., Shim J., Lee S., Hwang C.M., Durmus N.G., Demirci U. (2010). Microporous cell-laden hydrogels for engineered tissue constructs. Biotechnol. Bioeng..

[212] Yang C., Gao C., Wan Y., Tang T., Zhang S., Dai K. (2011). Preparation and characterization of three-dimensional nanostructured macroporous bacterial cellulose/agarose scaffold for tissue engineering. J. Porous Mater..

[213] Mancuso Nichols C.A., Guezennec J., Bowman J.P. (2005). Bacterial exopolysaccharides from extreme marine environments with special consideration of the Southern Ocean, sea ice, and deep-sea hydrothermal vents: A review. Mar. Biotechnol..

[214] Laurienzo P. (2010). Marine polysaccharides in pharmaceutical applications: An overview. Mar. Drugs.

[215] Sun C., Wang J.W., Fang L., Gao X.D., Tan R.X. (2004). Free radical scavenging and antioxidant activities of EPS2, an exopolysaccharide produced by a marine filamentous fungus *Keissleriella* sp. YS 4108. Life Sci..

[216] Sun C., Shan C.Y., Gao X.D., Tan R.X. (2005). Protection of PC12 cells from hydrogen peroxide-induced injury by EPS2, an exopolysaccharide from a marine filamentous fungus *Keissleriella* sp. YS4108. J. Biotechnol..

[217] Liu F., Ng T.B. (2000). Antioxidative and free radical scavenging activities of selected medicinal herbs. Life Sci..

[218] Schinella G.R., Tournier H.A., Prieto J.M., de Mordujovich Buschiazzo P., Ríos J.L. (2002). Antioxidant activity of anti-inflammatory plant extracts. Life Sci..

[219] Sun H.H., Mao W.J., Chen Y., Guo S.D., Li H.Y., Qi X.H., Chen Y.L., Xu J. (2009). Isolation, chemical characteristics and antioxidant properties of the polysaccharides from marine fungus *Penicillium* sp. F23-2. Carbohydr. Polym..

[220] Colliec-Jouault S., Zanchetta P., Helley D., Ratiskol J., Sinquin C., Fischer A.M., Guezennec J. (2004). Les polysaccharides microbiens d’origine marine et leur potentiel en thérapeutique humaine. Pathol. Biol..

